# Retinotopic Maps, Spatial Tuning, and Locations of Human Visual Areas in Surface Coordinates Characterized with Multifocal and Blocked fMRI Designs

**DOI:** 10.1371/journal.pone.0036859

**Published:** 2012-05-09

**Authors:** Linda Henriksson, Juha Karvonen, Niina Salminen-Vaparanta, Henry Railo, Simo Vanni

**Affiliations:** 1 Brain Research Unit, O.V. Lounasmaa Laboratory, Aalto University, Espoo, Finland; 2 Advanced Magnetic Imaging Centre, Aalto University, Espoo, Finland; 3 MRC Cognition and Brain Sciences Unit, Cambridge, United Kingdom; 4 Centre for Cognitive Neuroscience, University of Turku, Turku, Finland; 5 Department of Psychology, University of Turku, Turku, Finland; The University of Sydney, Australia

## Abstract

The localization of visual areas in the human cortex is typically based on mapping the retinotopic organization with functional magnetic resonance imaging (fMRI). The most common approach is to encode the response phase for a slowly moving visual stimulus and to present the result on an individual's reconstructed cortical surface. The main aims of this study were to develop complementary general linear model (GLM)-based retinotopic mapping methods and to characterize the inter-individual variability of the visual area positions on the cortical surface. We studied 15 subjects with two methods: a 24-region multifocal checkerboard stimulus and a blocked presentation of object stimuli at different visual field locations. The retinotopic maps were based on weighted averaging of the GLM parameter estimates for the stimulus regions. In addition to localizing visual areas, both methods could be used to localize multiple retinotopic regions-of-interest. The two methods yielded consistent retinotopic maps in the visual areas V1, V2, V3, hV4, and V3AB. In the higher-level areas IPS0, VO1, LO1, LO2, TO1, and TO2, retinotopy could only be mapped with the blocked stimulus presentation. The gradual widening of spatial tuning and an increase in the responses to stimuli in the ipsilateral visual field along the hierarchy of visual areas likely reflected the increase in the average receptive field size. Finally, after registration to Freesurfer's surface-based atlas of the human cerebral cortex, we calculated the mean and variability of the visual area positions in the spherical surface-based coordinate system and generated probability maps of the visual areas on the average cortical surface. The inter-individual variability in the area locations decreased when the midpoints were calculated along the spherical cortical surface compared with volumetric coordinates. These results can facilitate both analysis of individual functional anatomy and comparisons of visual cortex topology across studies.

## Introduction

Human cerebral cortex contains multiple orderly representations of the visual field. This retinotopic visual field topography is particularly clear in the early visual areas V1, V2, and V3, where it was evident already in the early brain imaging studies, but exists also in several higher-level visual areas (for reviews, see [1], [2], [3]). The retinotopic organization is the main criterion for delineation of several visual areas in the human cortex. Retinotopy is most commonly mapped using a periodic visual stimulus that moves across the visual field and produces a travelling wave of activity along the retinotopic cortex [4], [5], [6], [7]. With this phase-encoded (or travelling wave) method, several retinotopic maps have been identified in the medial occipital (V1–3) [4], [6], [8], ventral (hV4, VO1–2, PHC1–2) [9], [10], [11], dorsal occipito-parietal (V3A, V3B, V6, IPS0–4) [12], [13], [14], [15], [16], [17] and lateral occipito-temporal cortex (LO1–2, TO1–2, V5/hMT+) [18], [19], [20], [21].

The average receptive field size of neurons in a visual area affects the fMRI response evoked by a stimulus moving across the visual field [14], [22]. In higher-level visual areas, neurons on average have large receptive fields, and hence respond to a large portion of the visual field. Even then, if the receptive field centres are organized retinotopically and the signal-to-noise ratio of the measurement is good enough, the retinotopic map can be measured [23]. However, the fMRI mapping experiment must be carefully optimized to be able to map the retinotopic organization in a specific higher-level visual area [2], [23].

We have aimed to develop retinotopic mapping methods that employ the standard general linear model (GLM) implemented in any conventional software package for fMRI analysis. A straightforward approach for the localization of visual areas and retinotopic regions-of-interest is important in many imaging studies where the retinotopic organization, per se, is not of interest. This applies not only to fMRI studies, but also, for example, to transcranial magnetic stimulation (TMS) experiments. Here we describe two approaches for retinotopic mapping: a 24-region multifocal stimulus (multifocal mapping; an improved version of the method originally presented by Vanni et al. [24]) and a blocked presentation of object stimuli at different visual field locations (object mapping). Our first objective was to examine whether these GLM-based approaches can capture the polar angle and eccentricity maps in several visual areas in a reasonable data acquisition time. Previous studies using a blocked stimulus presentation have reported contralateral visual field preference but no detailed retinotopic organization in higher-level visual areas [25], [26], [27], where retinotopy is evident when mapped with the phase-encoded approach [16], [21], [28]. To complement the description of retinotopy across the hierarchy of visual areas, we introduced a measure for spatial tuning. The strength of the tuning was estimated based on how much each cortical location responded not only to the optimal stimulus region but also to the stimuli at other polar angles.

In addition to studying individual subjects, we were interested in the variability of the retinotopic cortex between subjects. The conventional volume-based spatial normalization of individual data to a standard brain atlas is typically considered an inappropriate approach for retinotopic visual areas, because of the large inter-individual variability in the size, shape and position of the areas [8], [29], [30]. Spatial normalization based on cortical surface yields to more accurate alignment results of functional areas located near specific sulci [31], [32], such as the primary visual cortex (V1) within the calcarine sulcus [32], [33]. Cortical surface-based analysis methods respect the sulcal topology of the cortical surface and provide also a coordinate system that describes cortical positions better than the conventional brain volume-based coordinates [32], [34], [35], [36]. More specifically, when a cortical hemisphere is transformed onto a sphere or an ellipsoid, nearby latitude and longitude coordinates refer to nearby cortical locations, which is not true for conventional 3D stereotaxic coordinates [31], [36]. Despite the attractiveness of the surface-based coordinates, positions of cortical areas and activation foci are still most commonly reported in the 3D stereotaxic coordinates. Here we studied the inter-individual consistency of the visual area positions in the spherical surface-based coordinate system implemented in the widely used Freesurfer software [31], [32]. We explored the longitude and latitude coordinates of each visual area and evaluated the variability of the individual visual area loci on a group-average cortical surface.

Finally, we constructed probability maps of the retinotopic visual areas, which can be used together with Freesurfer's surface-based atlas of the human cerebral cortex. This enables other studies using Freesurfer to assign their visual cortex activation to these probability maps of the visual areas. There is a growing interest in surface-based probabilistic atlases of the human brain, which aim to depict the probability of any functional area in a specific cortical location [33], [37], [38], [39], [40]. Hinds et al. [33], [38] showed that cortical folding predicts accurately the location of V1, but recently Yamamoto et al. [39] presented much lower average probabilities for several visual areas. We anticipated that on the cortical surface, the locations of the extrastriate visual areas are much more consistent between individuals than is generally assumed based on stereotaxic studies on the visual cortex.

## Methods

### Subjects

Fifteen subjects (S1–S15, ages 21–28, 8 females) participated in this study, which was approved by the local ethics committee of the Hospital District of Helsinki and Uusimaa. All subjects had normal or corrected-to-normal vision. The subjects gave written informed consent before participating in the measurements. For one subject (S1), the high-resolution anatomical, and the multifocal and object mapping data were collected successively within the same measurement session. For the others, a short break outside the scanner separated the multifocal (four 4-minute runs) and object (four 4-minute runs) mapping measurements.

### Experiments

#### Multifocal mapping

**Figure 1:**
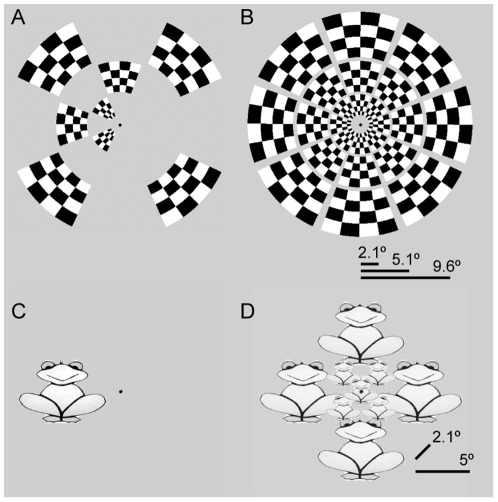
Stimuli for the multifocal and object mapping. The mapping tools can be obtained from the website http://ltl.aalto.fi/wiki/RetinotopicMapping. A) One frame of the 24-region multifocal stimulus. B) In the multifocal mapping, the visual field from 1° to 12° eccentricity was divided to 24 regions, which were stimulated in parallel with a contrast-reversing checkerboard pattern using temporally orthogonal stimulus sequences [24]. The subjects fixated a point in the middle of the stimulus. For a video excerpt of the multifocal stimulus, see Video S1 in the Supporting Information. C) One frame of the object stimulus. D) In the object mapping, nine regions in the visual field (fovea and eight different polar angles at two different eccentricities) were stimulated with object images using a blocked fMRI design. For a video excerpt of the object stimulus, see Video S2 in the Supporting Information.

With the multifocal stimulus the visual field (1°–12°) was divided into 24 regions, in 3 rings and 8 wedges (Fig. 1B, Video S1). The three rings extended eccentricities 1°–3.2°, 3.5°–6.7° and 7.2°–12°. The regions were stimulated with a high-contrast checkerboard pattern using temporally orthogonal stimulus sequences (for details on the quadratic residue sequences used to produce the temporally orthogonal time series, see Vanni et al [24]). The subjects passively fixated a point in the middle of the stimulus. Approximately half (10–15) of the 24 stimulus regions were stimulated during one miniblock (duration 7.2 seconds), but because the multifocal responses are affected by nonlinearities in spatial summation [41], these regions were stimulated in two temporally distinct sets. The regions were divided into these sets so that regions sharing a border were never stimulated at the same time. During a miniblock, the one set of regions was on for 115 ms+115 ms in two opposing contrasts (Fig. 1A), and then the checkerboard pattern disappeared for 135 ms, after which a second set of regions was displayed. The two sets of regions were shown multiple times during one miniblock with the stimulus-onset-asynchrony of 365 ms. Each multifocal run consisted of 33 miniblocks (total duration: 33×7.2 seconds = 3 min 57.6 sec) with no stimulation during the first and last miniblocks. To reach stable T1 magnetization, the data from the first miniblock was excluded from the data analysis. Altogether four experimental runs with multifocal stimulation were measured for each subject.

### Object mapping

During the object mapping, grayscale images of objects were presented at nine different visual field locations (altogether three eccentricities and eight polar angles; Fig. 1D, Video S2) in a block design. The 50 different objects were extracted from photographs obtained from free online photograph libraries (http://www.freeimages.co.uk, http://www.morguefile.com). The stimuli on the vertical and horizontal meridians subtended on average the eccentricities 2°–8° (diameter of the stimuli) with a mean eccentricity of 5° and the stimuli on the oblique polar angles the eccentricities 0.8°–3.5° with a mean of 2.1°. The stimuli were presented at the meridians and at the oblique polar angles in separate experimental runs. Within an experimental run, different object images (30 in one block) were presented consecutively for 14.4 seconds at one position (Fig. 1C). Rest blocks (7.2 seconds) separated the five different stimulus blocks (fovea and four polar angles). Two repetitions of each stimulus position were measured within one experimental run, total duration: 2 [repetitions]×5 [stimulus positions]×(14.4 seconds [stimulus block duration]+7.2 seconds [rest block duration])+7.2 seconds [rest block in the beginning]+14.4 seconds [rest block in the end] = 3 min 57.6 sec. The first four time points (7.2 seconds) from the beginning of each run were excluded from the analysis. The subjects passively fixated a point at the center of the screen throughout the experimental runs. Two experimental runs were measured for each condition (meridians and oblique polar angles). The stimuli centred at the fovea were of two different sizes in the two conditions, but only the responses for the smaller foveal stimulus (shown in Fig. 1D), mapped together with the oblique polar angle stimuli, were used in the retinotopic maps. The order of the stimulus positions was pseudo-randomized and balanced within the experiment.

#### Visual motion localizer

Cortical areas sensitive to visual motion were localized with a separate functional localizer. The localization was based on the comparison between responses to moving and stationary low-contrast rings. In the movement condition, the concentric rings expanded or contracted at 7°/s. For 14 subjects the motion localizer was measured together with the multifocal mapping and for one subject (S5) together with the object mapping.

### Stimulus setup

All stimuli were created with Matlab™ (Mathworks, Natick, MA) and their timing was controlled with Presentation™ (Neurobehavioral Systems, Albany, CA). The stimuli were projected with a 3-micromirror Christie X3™ (Christie Digital Systems, Kitchener, Ontario, CA) data projector to a semitransparent screen, which the subjects viewed via a mirror at a 34 cm distance.

### Data acquisition and analysis

FMRI measurements were performed with a 3T GE Signa scanner (General Electric Medical Systems, Milwaukee, WI, USA) with HDxt update and an 8-channel receiver head coil. Functional volumes (voxel size: about 3 mm×3.1 mm×3.1 mm) were acquired with echo planar imaging using single-shot gradient-echo sequence with imaging parameters: repetition time 1.8 s (multifocal and object mapping) or 2.0 s (motion localizer), 29 slices with 3.0 mm slice thickness (no gap), field of view 20 cm, imaging matrix 64×64, echo time 30 ms, and flip angle 60°. Two sets of high-resolution T1-weighted anatomical images (voxel size: 1 mm×1 mm×1 mm) were acquired with 3D SPGR BRAVO-sequence with ASSET calibration and acceleration with a factor of two. For each subject, the white and gray matter borders were segmented and reconstructed from the anatomical images using the Freesurfer software package [32], [42].

Functional data were analyzed with SPM8 (Wellcome Department of Imaging Neuroscience, London, UK) Matlab™ toolbox (for an overview, see [43]). In preprocessing, functional images were corrected for interleaved acquisition order and for head motion. To preserve spatial resolution, no spatial smoothing was applied. In statistical analysis, the timing of the stimulus blocks were entered as regressors of interest to the general linear model and convolved with the canonical hemodynamic response model. Parameters describing head motion were included as nuisance variables (covariates of no interest). During the parameter estimation, the data were high-pass filtered with a 128-s cut-off, and serial autocorrelations were estimated with restricted maximum likelihood algorithm using a first-order autoregressive model. Estimates for the fMRI % signal changes for each stimulus within each voxel were calculated from the parameter estimate images.

### Retinotopic maps

The multifocal and object mapping data were converted to eccentricity and polar angle maps using weighted averaging of the responses. A similar approach has been previously used by Hansen et al [44].The eccentricity maps were constructed by calculating a weighted eccentricity value 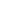 for each voxel
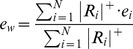
(1)where 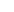 is the number of stimulus regions (24 for multifocal mapping and 9 for object mapping), 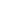 are the fMRI % signal changes for the different stimuli 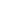 and 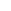 are the eccentricities of the different stimuli 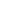. 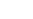 denotes that only positive responses were allowed, *i.e.*, the negative responses were set to zero. Negative responses were ignored, because they are typically observed in cortical locations surrounding the actual retinotopic representation of the stimulus and reflect long-range mechanisms, such as surround suppression [45], [46]. In addition, voxels in which the response for none of the stimuli exceeded a t-value threshold of 3 were excluded to reduce noise in the retinotopic maps.

The polar angle maps were constructed by calculating a weighted polar angle value 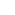 for each voxel. Because polar angle is a circular quantity, the weighted polar angle was estimated using weighted vector averaging
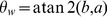
(2)where 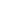 and 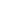 are
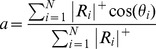
(3)

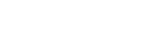
and 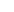 is the number of stimulus regions (24 for multifocal mapping and 8 for object mapping), 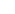 are the fMRI % signal changes for the different stimuli 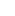 and 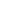 are the polar angles of the different stimuli 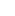. Again, negative responses were set to zero, and voxels with t-values less than 3 for all stimulus regions were excluded from the maps.

### Spatial tuning

We complemented the analysis of retinotopy by examining the spatial tuning in different visual areas from the object mapping data. We visualized spatial tuning curves in which the fMRI % signal change was plotted as a function of the polar angle of the object stimuli. To quantify the strength of the spatial tuning in different visual areas, we estimated the strength of the polar angle tuning for each voxel using a vector averaging approach [47]

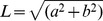
(4)This measure for tuning strength can be used for circular quantities (*e.g.*, orientation or polar angle), assuming that the test angles are distributed uniformly over the range of possible angles (here, the polar angles were 0°, 45°, … 315°) and the responses are ≥0 (here, negative responses were set to zero). The analysis was restricted to voxels, which exceeded a t-value threshold of 3 for any stimulus region.

### Group-average retinotopic maps

The retinotopic maps were averaged across the group of subjects using the cortical surface-based coordinate system implemented in the Freesurfer software [31], [32]. For each individual, the reconstructed cortical surface was inflated to a sphere, which was morphed into register with the Freesurfer's spherical atlas of the human cerebral cortex (Freesurfer's fsaverage subject, sphere.reg surface) based on the cortical curvature information. The individual data were resampled onto this average surface. Nodes with data from less than three subjects were omitted from the average retinotopic maps.

### Mean positions and distances on spherical cortical surface

The mean positions of the visual areas were reported in the spherical cortical surface-based coordinate system implemented in Freesurfer. Visual areas were manually labelled on individual cortical surfaces based on the retinotopic data and morphed on to the average cortical surface via the spherical transformation [31], [32]. The mean position of a visual area label was calculated on the average spherical cortical surface. Mean position 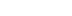 of N points 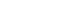 on a spherical surface expressed in Cartesian coordinates is calculated as
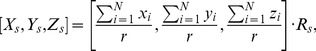
(5)where 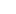 is the radius of the sphere and
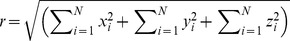
(6)


The inter-individual variability of the visual area positions was studied by evaluating the mean distances of the individual positions to the group-average position. The central angle 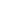 between two points on a sphere reflects the distance between them along the spherical cortical surface and can be calculated with the spherical law of cosines
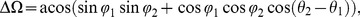
(7)where 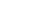 and 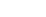 are the latitude and longitude coordinates of the two points. The distance along the spherical surface is then 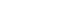, where 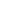 is the radius of the sphere.

### Construction of the surface-based probabilistic maps

Probability maps of the visual areas were constructed by counting the number of times a node in the cortical surface was labelled as a specific visual area (maximum 15) and by dividing this with the number of subjects (15). The visual area labels were drawn manually on the individual's cortical surface based on the retinotopic data and brought into the average cortical surface using the spherical surface-based alignment. Each label from each subject was confined to nodes with polar angle data from that subject. To improve inter-individual alignment, the data were smoothed by a 1 mm kernel along the cortical surface. The effect of the smoothing was merely to reduce cortical nodes labelled as non-visual by filling holes in the labels. In addition, nodes with data from less than three subjects were omitted from the probability maps.

## Results

### The applicability of the multifocal and blocked fMRI designs for retinotopic mapping

**Figure 2:**
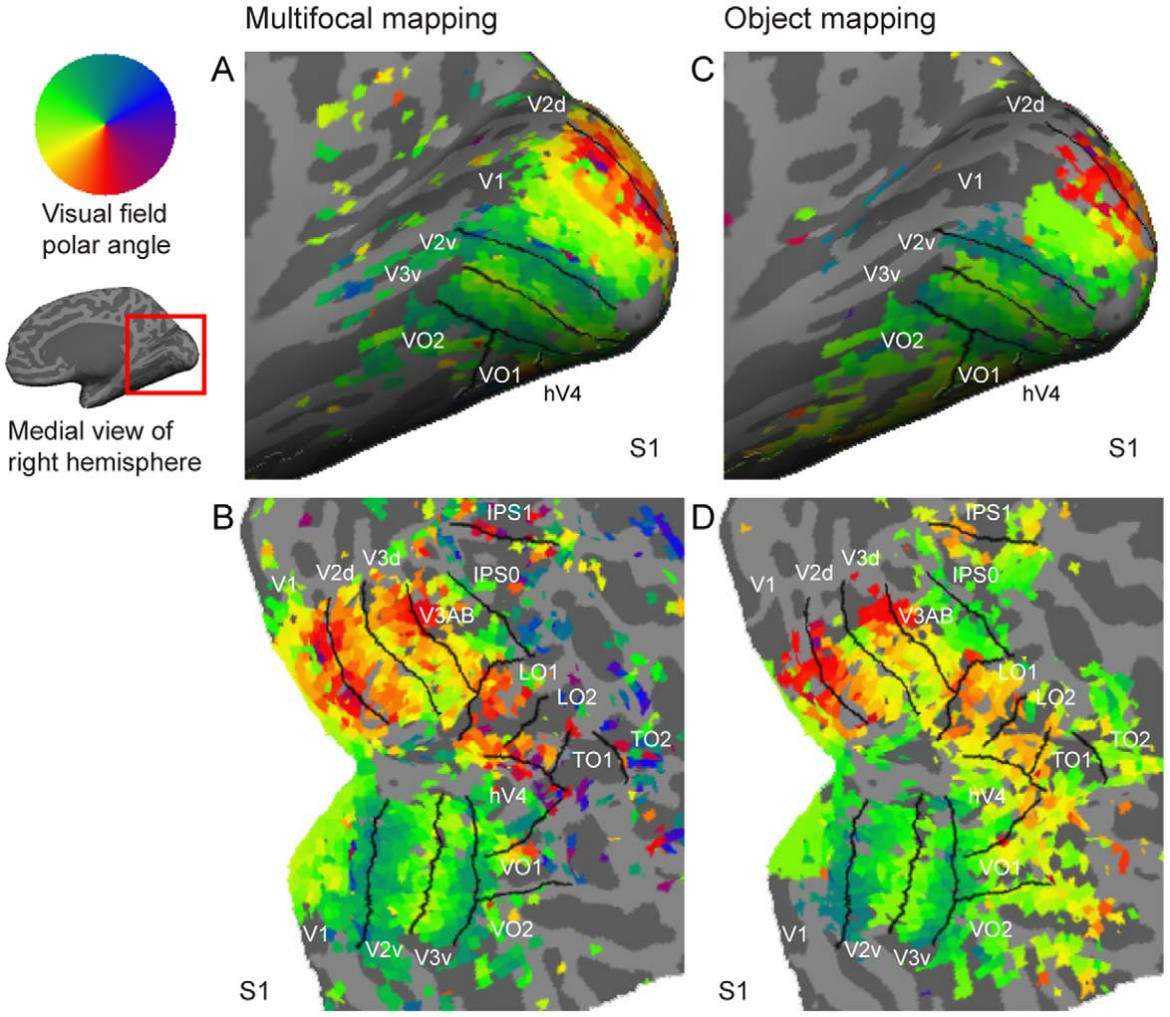
Representative retinotopic maps obtained with the multifocal and object mapping. A) Medial view and B) unfolded patch of the subject's right occipital cortical surface show the polar angle map obtained with the 24-region multifocal stimulus. C) Medial view and D) unfolded patch of the subject's right occipital cortical surface show the polar angle map obtained with the 9-region object stimulus. See Supplementary Figures S2, S3, S4, S5 for the polar angle and eccentricity maps from both mapping measurements for all 15 subjects.

To demonstrate that our stimulus designs are suitable for retinotopic mapping of the visual cortex, we compared the retinotopic maps obtained with the two approaches and made comparisons with the phase-encoded retinotopic data described in the literature. Figure 2 shows representative retinotopic maps obtained with the multifocal and the object mapping. Results for all 15 subjects from both hemispheres are presented as supplementary material (Figs. S2, S3, S4, S5). The polar angle maps were constructed using the weighted vector averaging of the responses (Eq. 2). The retinotopy in visual areas V1, V2 and V3 could be mapped with both stimulus designs, but the multifocal method with finer sampling of the visual field with the 24 stimulus regions mapped the retinotopic representations with higher fidelity than the 9-region object stimulus. The multifocal maps also extended further in the peripheral representation of the visual field than the maps obtained with the object stimulus, because the multifocal stimulus (Figs 1A, B) covered the visual field up to 12° and the object stimulus (Figs 1C, D) up to approximately 8°. In the intermediate visual areas V3AB and hV4, the multifocal and object measurements produced comparable maps, but in the higher-level areas, as discussed below in more detail, the multifocal responses were greatly reduced and the multifocal maps were of inferior quality compared to the object mapping results.

Our efforts to map the retinotopic organization in the higher-level visual areas with modified spatial (larger stimulus regions, images of objects instead of checkerboards) and temporal parameters of the multifocal stimulus have failed (see Supplementary Figure S1 for representative results from pilot experiments). We had anticipated beforehand that multifocal responses are affected as response nonlinearities increase in higher-level visual areas [41], [48], but it was still surprising to find out that we could not measure the multifocal responses in the higher-level visual areas even when the number of stimulus regions was reduced to as few as five and the checkerboards were replaced with images of objects. This suggests that the spatial and temporal summation of information across different locations of the visual field is highly nonlinear in the higher-level visual areas, and linear analysis methods are probably not suited to recover the concurrent activation of multiple activation patterns. With the blocked presentation of the object stimuli, the retinotopic maps could be identified in several higher-level visual areas (Fig. 2D): ventral occipital areas 1 and 2 (VO1, VO2), intraparietal sulcus area 0 (IPS0), lateral occipital areas 1 and 2 (LO1, LO2) and temporal occipital areas 1 and 2 (TO1, TO2). A more detailed description of the retinotopic organization in the higher-level visual areas follows.

**Figure 3:**
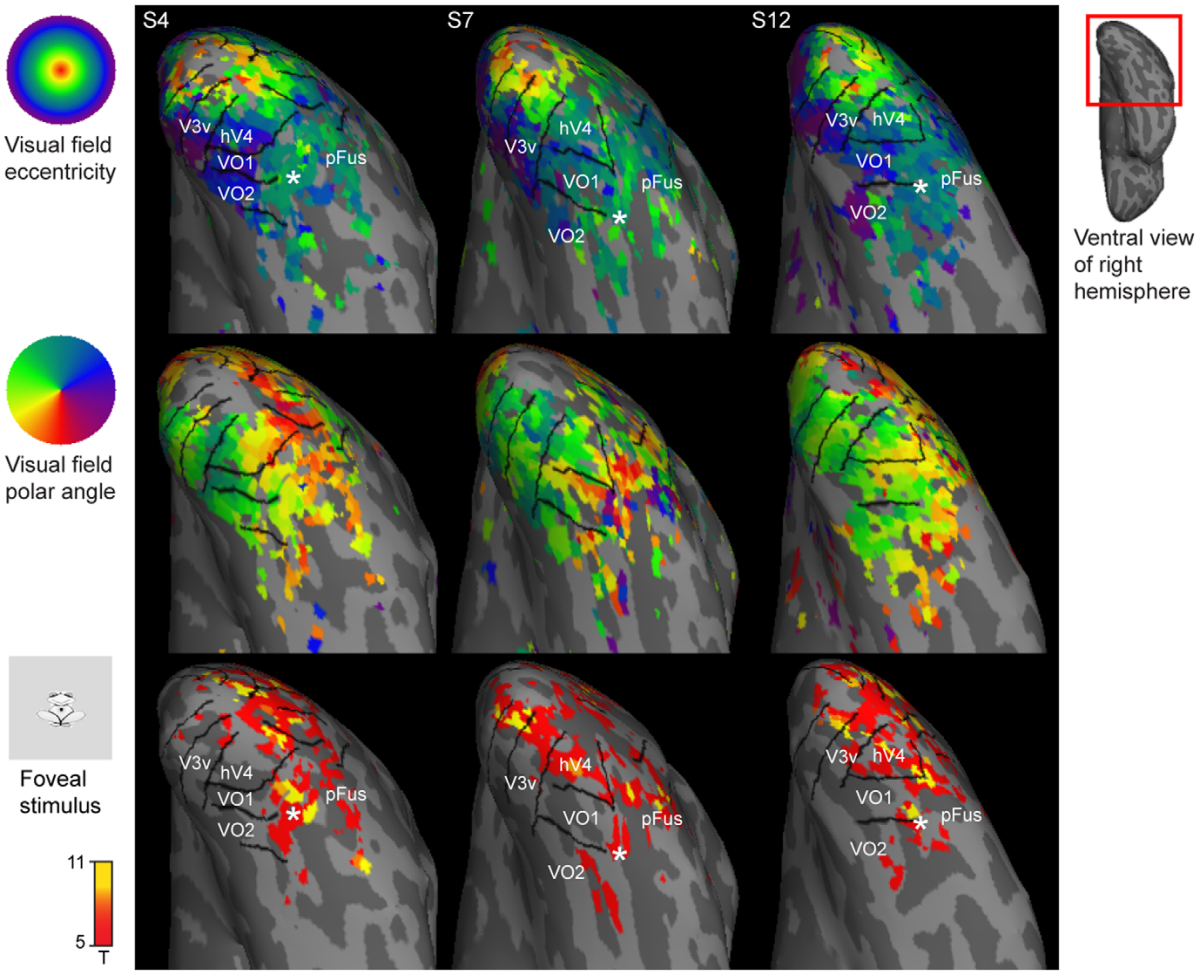
Retinotopic organization of the ventral visual cortex. The top panel shows the retinotopic eccentricity maps and the middle panel the retinotopic polar angle maps obtained with the object mapping for representative subjects S4, S7, and S12. The bottom row shows the activation pattern for the foveal object stimulus. The distinct representations of the fovea in areas VO1 and VO2 are also denoted by asterisks on the eccentricity maps. See Supplementary Figures S6 and S7 for the maps for all subjects and for both hemispheres.

Figure 3 shows ventral views of the retinotopic maps obtained with the object mapping (blocked stimulus design) for three representative subjects. Results for all 15 subjects from both hemispheres are presented as supplementary material (Figs. S6 and S7). The eccentricity maps were constructed using the weighted averaging of the responses (Eq. 1) and the polar angle maps using the weighted vector averaging of the responses (Eq. 2). Consistent with the previous studies using phase-encoded or population receptive field retinotopic mapping stimuli [9], [10], [13], the retinotopic map in hV4 was shorter than the maps in areas V1–V3 and, in most subjects, represented the full contralateral hemifield. At the border between hV4 and VO1 was a representation of the visual periphery (blue/purple in the eccentricity maps) and the representation of the lower vertical meridian typically curved towards the V3v/hV4 border (orange/red in the polar angle maps, for examples, see Fig. 3).

The representation of the fovea in areas VO1 and VO2 is distinct from the confluent foveal representation in the early visual areas and the eccentricity map runs from this foveal representation towards area V3v [13]. Overall VO1 and VO2 maps are relatively small and have strong cortical magnification [13], and thus there is more overlap between the representations of the stimulus regions at the current spatial resolution compared to activation patterns in the early visual cortex (see also following results on spatial tuning). This overlap blurs the eccentricity maps constructed using weighted averaging, and therefore the colour range in our VO eccentricity maps runs only from green to purple and not from red to purple as in the early visual areas. The activation pattern for the foveal stimulus is shown separately in Figure 3 to show the distinct representation of the fovea along the ventral surface. The relatively large foveal stimulus (Fig. 1D) evoked quite extensive activation. The border between VO1 and VO2 is characterized with a representation of the upper vertical meridian (green in the polar angle maps). Areas hV4 and VO1 could be identified in all 15 subjects in both hemispheres and area VO2 (including the outer boundary of area VO2 characterized with lower vertical meridian representation) in 12/30 hemispheres.

**Figure 4:**
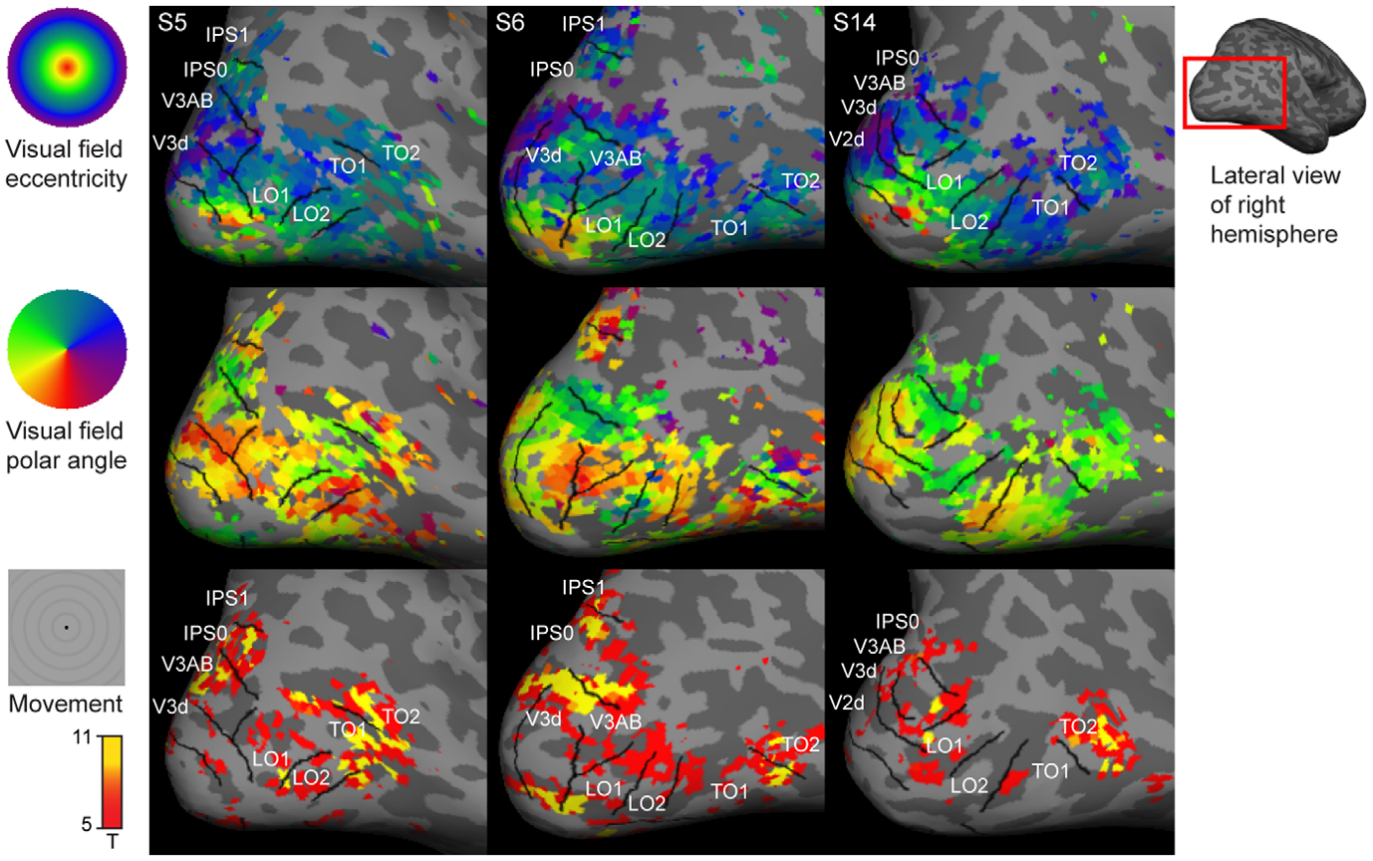
Retinotopic organization of the lateral visual cortex. The top panel shows the retinotopic eccentricity maps and the middle panel the retinotopic polar angle maps obtained with the object stimuli for representative subjects S5, S6, and S14. The bottom panel shows the cortical areas sensitive to visual motion. See Supplementary Figures S8 and S9 for the maps for all subjects and for both hemispheres.

Figure 4 shows lateral views of retinotopic maps obtained with the object mapping for three representative subjects. Results for all 15 subjects from both hemispheres are presented as supplementary material (Figs. S8 and S9). Our results are consistent with the LO1–2 [21] and TO1–2 [18] maps identified with the phase-encoded retinotopic mapping. They extend the previous blocked fMRI design studies that have reported merely a contralateral visual field preference without a retinotopic organization within the lateral occipital cortex [26], [27]. Area LO1 represented in most subjects the full contralateral hemifield and shared its posterior border with area V3d (yellow/red in the polar angle maps). The border between areas LO1 and LO2 was characterized by a representation of the upper vertical meridian (green in the polar angle maps). In most subjects, area LO2 also represented the full contralateral hemifield and may have a bias towards larger visual field eccentricities than visual area LO1. A representation of the lower vertical meridian marked the border between areas LO2 and TO1, and a representation of the upper vertical meridian defined the border between areas TO1 and TO2, which both represented the full contralateral hemifield. Figure 4 shows also the cortical areas that are sensitive to visual motion. Consistent with the results by Amano et al. [18], the TO1 and TO2 most likely correspond to the visual motion sensitive V5 complex [19], [49]. The retinotopy within the TO maps was not as clear as it was with the other areas, but we followed here the definition of the TO maps given by Amano et al. [18]. A more detailed view of the retinotopic organization could be possible with higher spatial resolution and signal-to-noise ratio [20]. We identified the retinotopic maps in LO1 in all 30 hemispheres, LO2 in 28/30 hemispheres, TO1 in 29/30 hemispheres, and TO2 in 27/30 hemispheres. In addition, the retinotopic maps in V3AB and IPS0 could be consistently identified with the object mapping stimuli. Area V3AB is located adjacent to area V3d and represents the full contralateral visual hemifield. IPS0 is located anterior to V3AB and also contains a full hemifield representation.

### Surface-based group-averages of the retinotopic maps

**Figure 5:**
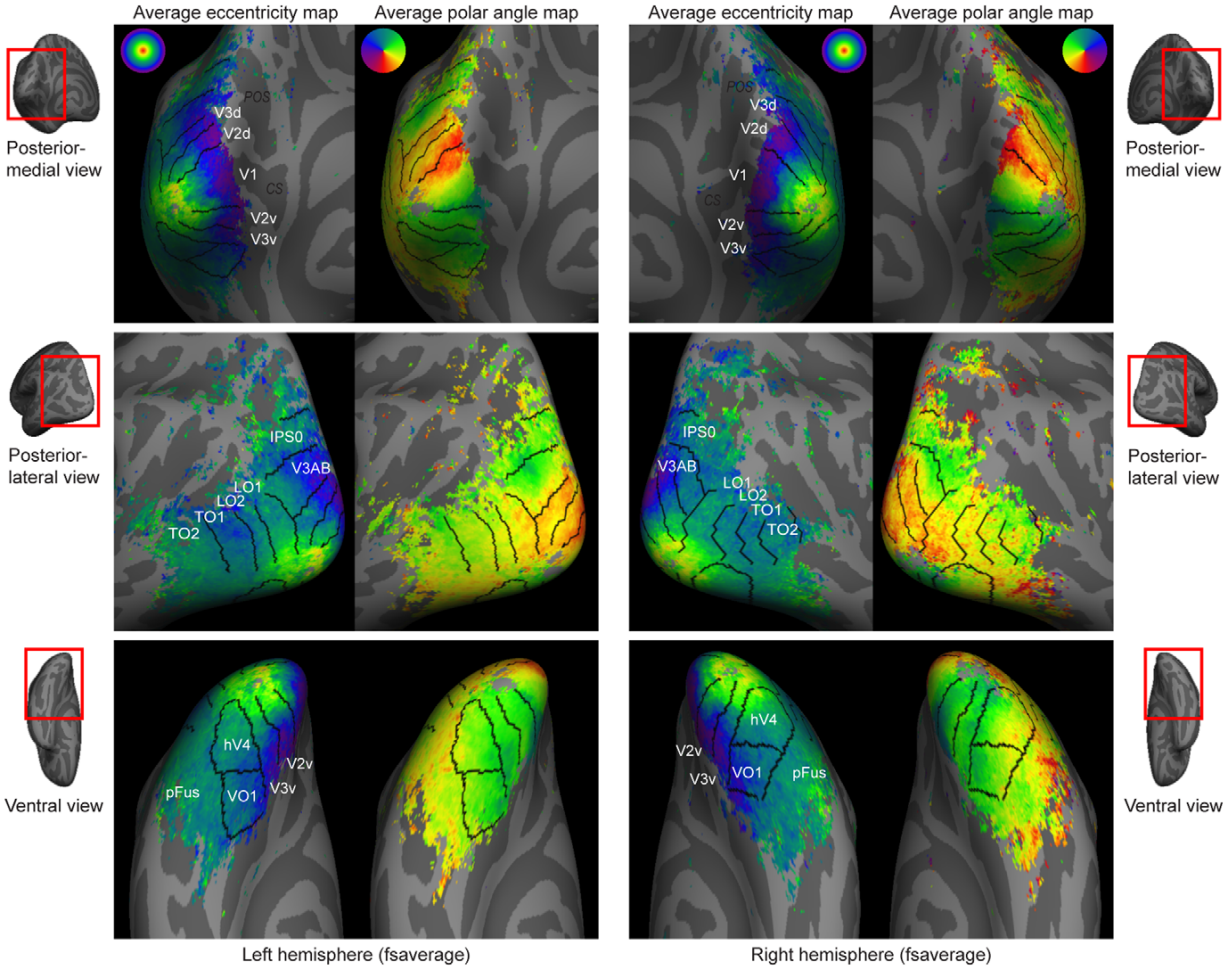
Group-average eccentricity and polar angle maps. The retinotopic maps obtained with the object mapping were averaged across the 15 subjects using the cortical surface-based coordinate system. Nodes with data from less than three subjects were omitted from the maps. The visual area borders were defined based on group-averages of the individuals' visual area labels brought into the average surface.

Figure 5 shows the retinotopic maps from the object mapping experiment averaged across the subjects using the cortical surface-based coordinate system [31], [32]. The borders between visual areas were drawn based on individual visual area labels averaged on the average surface. These area borders were consistent with the average retinotopic organization in the visual areas V1, V2, V3, V3AB, IPS0, hV4, and VO1. In lateral occipito-temporal cortex, the average retinotopic map was blurred, which implied greater inter-individual variability between the retinotopic organization and sulcal landmarks. This is evident also in Figures 3 and 4, where the orientation of the retinotopic maps in the lateral occipito-temporal cortex appeared to vary between the individuals more than the retinotopic organization along the ventral occipital cortex. For example, as seen in Figure 3, the polar angle maps appeared continuous between areas LO2 and hV4 in subject S4 whereas in subjects S7 and S12 there was a gap between them. Consistent with this, in a subset of subjects (*e.g.*, subject S5 in Figure 4) the polar angle representation was discontinuous between areas LO2 and TO1 whereas in the others it was continuous (*e.g.*, subject S6 in Figure 4).

**Figure 6:**
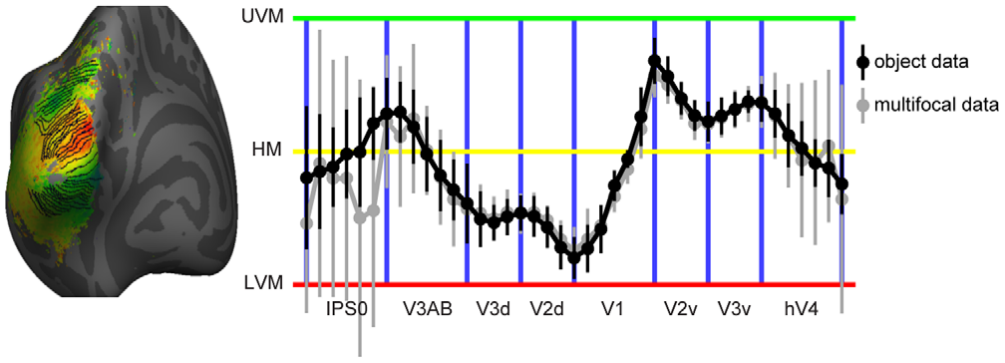
Iso-polar region-of-interest (ROI) analysis of the retinotopic organization. ROIs were drawn manually along lines of equal polar angle value on the group average retinotopic maps in the left and right hemispheres. The individual multifocal (gray markers) and the object (black markers) data were sampled by the same line ROIs drawn on the average surfaces. The data from the two hemispheres was averaged (UVM = upper vertical meridian; HM = horizontal meridian; LVM = lower vertical meridian). The error bars indicate the standard deviations of the polar angle values averaged across the subjects (N = 15).

The retinotopic organization on the average cortical surface was studied further using line-ROI analysis [11], [20]. This analysis complements the visual inspection of the retinotopic maps by showing the progression of the polar angle along the cortical surface. The line ROIs are drawn on the polar angle map, approximately parallel to the progression of the polar angle value, and then the values are collapsed across the different eccentricities. Here the isopolar line ROIs were drawn manually on the average polar angle map from the object mapping experiment (Figure 5) and both the multifocal and the object mapping data for each individual were sampled with the same ROIs on the average surface. The results are shown in Figure 6. The line ROI analysis verified the good correspondence between the multifocal and the object mapping data, especially in the areas V1, V2 and V3. Overall the profile of the polar angle progression across visual areas is very similar to the result shown by Larsson and Heeger [21] for phase-encoded retinotopic mapping data. In the lateral occipito-temporal cortex, there was too much inter-individual variability in our data for the line-ROI analysis on the average cortical surface.

### Visual areas differ in spatial tuning

**Figure 7:**
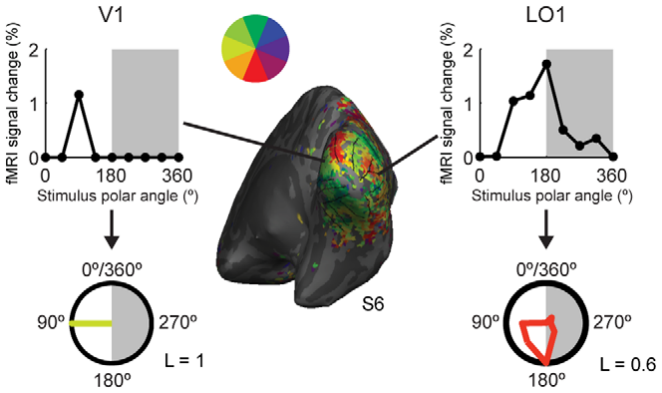
Spatial tuning curves. Representative single voxel tuning curves illustrate differences in the spatial tuning across visual areas in right hemisphere. The mean fMRI % signal changes are plotted as function of the polar angle of the object stimuli. The same data are also shown as polar plots, where the distance from the centre of the circle reflects the response amplitude at each polar angle and the colour codes the weighted average visual field position (see the colour wheel). The gray background highlights the responses for stimuli in the ipsilateral visual field. In the representative voxel within area V1, only the stimuli at the left horizontal meridian evoked a measurable response, whereas a range of stimulus positions produced measurable responses in the representative LO1 voxel. The L values are estimates for the strength of the spatial tuning (Eq. 4).

In typical retinotopic mapping, one estimates for each cortical location only the visual field position that is most effective in eliciting a response. Here we used an alternative approach as we combined responses across the stimulus regions (Eqs. 1 and 2). We assumed that especially in higher-level visual areas a large portion of the visual field could elicit a strong response and hence selecting only the most effective visual field position among a fixed number of sampled positions would not be the optimal approach. Figure 7 shows examples of spatial tuning curves, that is, plots of the mean fMRI signal change as a function of the polar angle of the object stimuli. The representative voxel in V1 responded only to one of the stimulus positions (left horizontal meridian), whereas the representative voxel in area LO1 responded to stimuli in any location within the left lower visual field quadrant and to a lesser extent also to stimuli in the right (ipsilateral) lower visual field quadrant. The same tuning curves are also shown as polar plots, where the distance from the centre of the circle reflects the response amplitude at each polar angle. The polar plots nicely visualize how the spatial tuning curves differ between visual areas. A more comprehensive view on visual field representations in different visual areas is thus achieved by considering for each voxel not only the most effective visual field location nor only the mean of the effective visual field locations but also the strength of the spatial tuning.

**Figure 8:**
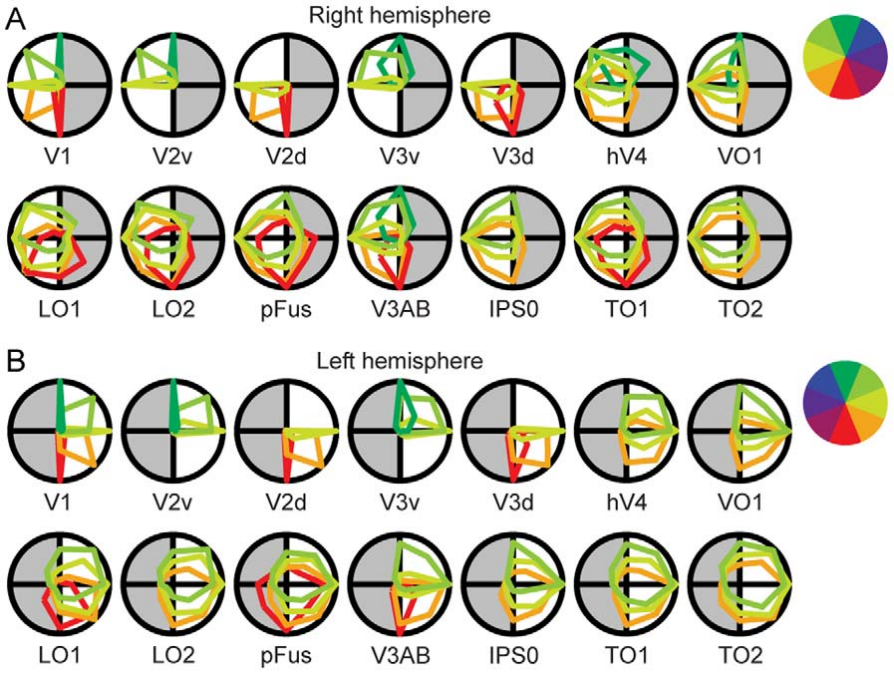
Polar plots of average spatial tuning curves obtained with the object mapping. A) Voxels within each visual area in the right hemisphere were classified to eight different classes according to the polar angle they represent. The colour indicates the polar angle. The polar plots illustrate how much each of these classes represents also other visual field positions. For example, in V1 the voxels representing the lower vertical meridian (shown in red) did not respond to stimuli at any other polar angle, whereas the V3d voxels that represented the lower vertical meridian did respond to some amount also to the stimuli at neighbouring locations, and the TO1 voxels that represented the lower vertical meridian responded at some amount to any of the stimuli. The tuning curves were first averaged across voxels within a visual area and then across the subjects. The grey background indicates the hemifield ipsilateral to the studied hemisphere. B) Same as in A for the visual areas in the left hemisphere.

Figure 8 shows the polar plots of average spatial tuning for different visual areas. The averaging was done separately for voxels classified to eight different groups according to the visual field location they represented (see the colour wheel in Fig. 8). In all areas, the “preferred” visual field position was in the contralateral visual field (no bluish colours in the plots). In the higher-level areas, however, the broad tuning curves covered also parts of the ipsilateral visual field. Interestingly, in an area around the posterior fusiform gyrus (pFUS), there was no clear retinotopic map (Fig. 3 and Supp. Figs. S4, S5, S6, S7), but the average tuning curves (Fig. 8) showed sensitivity to visual field position.

**Figure 9:**
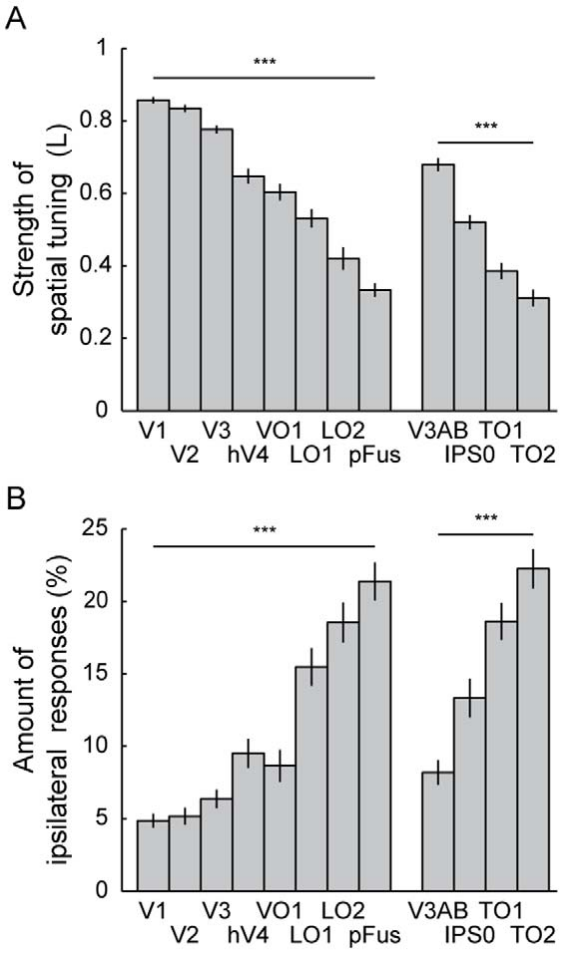
Strength of spatial tuning and amount of ipsilateral responses. A) The mean strength of the spatial tuning in different visual areas was averaged across the 15 subjects. The error bars indicate the standard errors of the means (SEMs) across the subjects. Visual area had a significant effect on the tuning strength (***p<0.001, Page's L test for the trends). B) The mean amount of ipsilateral responses was defined as the sum of responses for stimuli in the ipsilateral visual field divided by the sum of responses for all of the stimuli. Negative responses were ignored. The results were averaged across the 15 subjects and the error bars indicate the SEMs across the subjects. Visual area had a significant effect on the amount of ipsilateral responses (***p<0.001, Page's L test for the trends).

**Figure 10:**
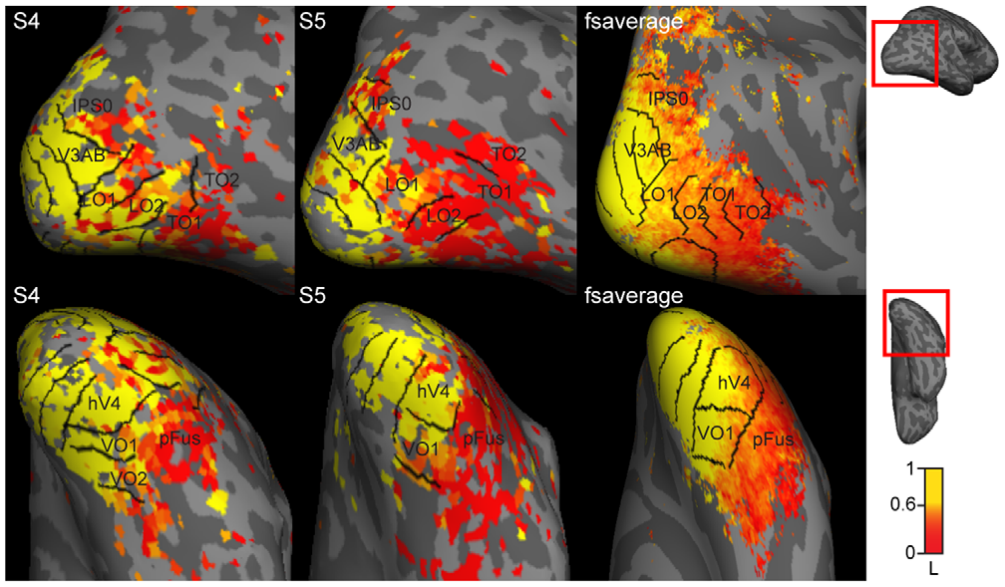
Cortical maps of spatial tuning strength. Lateral and ventral views of spatial tuning strength maps for two representative subjects (S4, S5) and a group-averaged tuning strength map. See Supplementary Figure S10 for data for all subjects and for both hemispheres.

We estimated the strength of spatial tuning for each visual area by calculating the strength of the polar angle tuning for each voxel (Eq. 4), averaging the values in the two hemispheres, and finally, averaging the results across the subjects. The results are shown in Figure 9A. Visual area had a significant effect on the tuning (Friedman test, p<0.001). The tuning strength decreased significantly along the hierarchy of visual areas in the ventral stream (V1 – pFus: Page's L test, L = 3045, p<0.001) and in the putative dorsal stream areas (V3AB – TO2: Page's L test, L = 416, p<0.001 [subject S13 excluded because of unclear TO maps]). The lower tuning strength implies broader spatial tuning, i.e., that on average a single voxel within an area responds to a wider range of polar angles. The tuning strength can also be visualized on the cortical surface. Figure 10 shows the results for two representative subjects and the surface-based group-average of the tuning strength map in the right hemisphere. The individual tuning strength maps from both hemispheres for all 15 subjects are presented in the Supplementary Figure S10. Note the clear transition in the tuning strength in the border of visual areas hV4 and pFus in the individual data. The difference in the strength of the spatial tuning between the early and higher-level visual areas is obvious also in the group-averaged map. Thus, the tuning strength data seems to be useful additional information for delineating pFUS from hV4 and VO1.

Figure 9B shows the amount of ipsilateral responses in each visual area. This was calculated for each voxel contributing to the polar angle maps as the sum of responses for the object mapping stimuli in the ipsilateral visual field divided by the sum of responses for all stimuli (excluding foveal stimuli). For the left/right hemisphere the ipsilateral stimuli were the three stimulus locations (horizontal meridian + two oblique polar angles) in the left/right hemifield. As with the other calculations, negative responses were set to zero. Visual area had a significant effect on the amount of ipsilateral responses (Friedman test, p<0.001). There were significant trends towards stronger representation of the ipsilateral visual field along the hierarchy of visual areas in the ventral stream (V1 – pFus: Page's L test, L = 2984, p<0.001) and in the putative dorsal stream areas (V3AB – TO2: Page's L test, L = 412, p<0.001 [subject S13 excluded because of unclear TO maps]). Note that while the proportion of ipsilateral responses was significant in the higher-level visual areas, the responses to the stimuli in the contralateral hemifield dominated in all studied areas.

### Anisotropies in visual field representations

**Figure 11:**
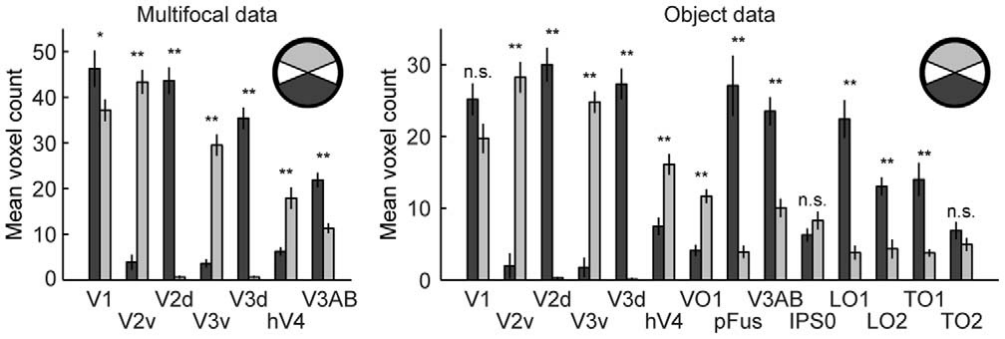
Asymmetries in visual field representations. The mean numbers of voxels that represented the lower (dark gray bars) and the upper (light gray bars) visual fields in different visual areas were averaged across the 15 subjects. The error bars indicate the SEMs across the subjects. **p<0.005, Wilcoxon Signed Rank-test; n.s., not significant (p>0.05, Wilcoxon Signed Rank-test).

To quantify how uniformly visual areas represent the visual field, we compared the mean number of voxels representing upper and lower visual fields. Figure 11 shows that areas hV4 and VO1 showed an overrepresentation of the upper visual field, whereas areas LO1, LO2, pFus, V3AB and TO1 overrepresented the lower visual field (Wilcoxon Signed Rank-test; see Fig. 11: **p<0.005 or *p<0.05, for each area). There was also a tendency towards a lower visual field bias in the early visual areas (ventral and dorsal divisions combined). However, this tendency was statistically significant only in the multifocal data in areas V1 and V3 (Wilcoxon Signed Rank-test, *p<0.05, for both areas).

### Locations of visual areas in spherical surface-based coordinate space

The mean locations of functional areas are commonly reported in some standard coordinate space to facilitate comparisons across studies. For this purpose the individual data are normalized to a brain atlas. The large inter-individual variability in the positions, sizes and shapes makes the volume-based spatial normalization of visual areas difficult [8], [29], [30]. In addition, because the topology of the visual cortex follows the cortical surface instead of the volumetric brain space, a 2D cortical surface-based coordinate system [31], [34], [36] should represent the visual area positions better than a 3D stereotaxic coordinate system. Therefore we wanted to characterize the mean and variability of the visual area positions in the spherical cortical surface-based coordinate system provided by Freesurfer [31].

**Figure 12:**
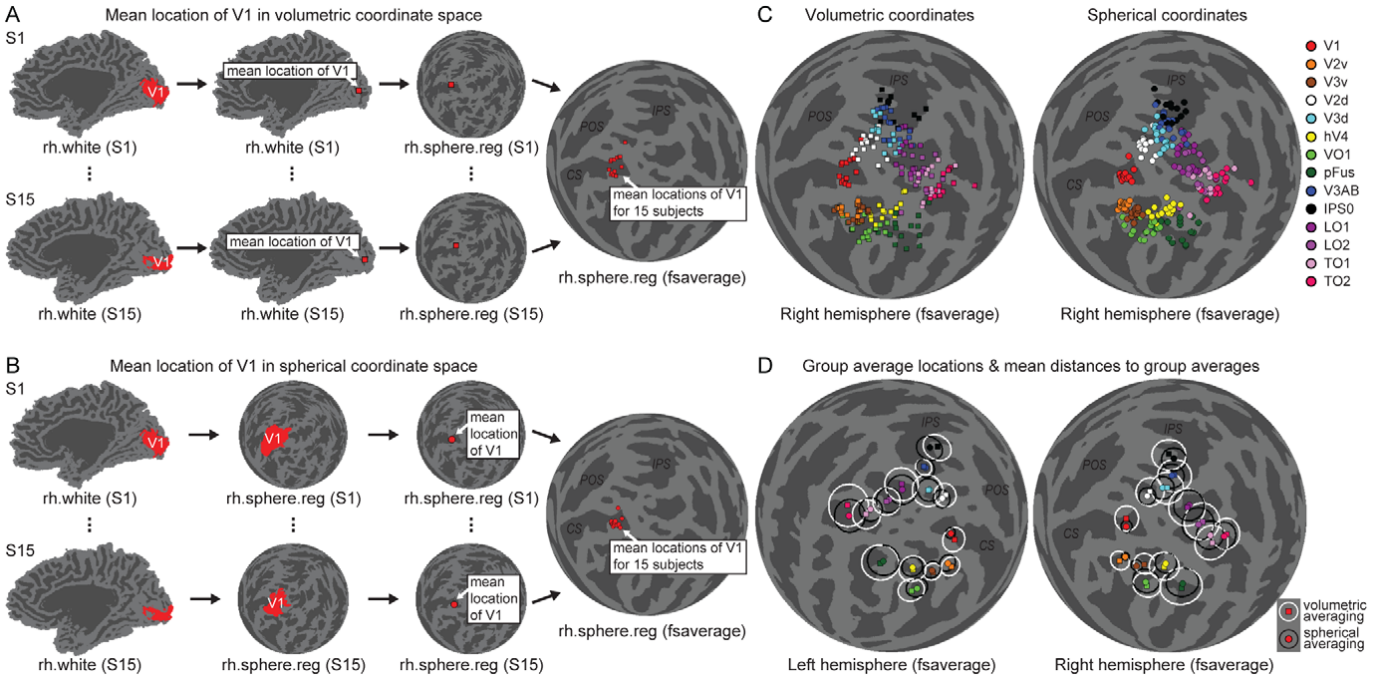
A comparison of calculating the midpoint of an area in volumetric or spherical coordinate system. A) An illustration of the clustering of the mean locations of area V1 for 15 subjects on the average cortical surface when the mean location of area V1 was calculated for each subject in the volumetric (cartesian) coordinate system. Note the spread of the points along the lips of the calcarine sulcus (CS = Calcarine Sulcus; POS = Parieto-Occipital Sulcus; IPS = Intra-Parietal Sulcus). B) An illustration of the clustering of the mean locations of area V1 for the 15 subjects on the average cortical surface when the mean location of area V1 was calculated for each subject along the cortical surface-based spherical coordinate system. Note the clustering of the points at the base of the calcarine sulcus. C) The mean locations of several visual areas for 15 subjects calculated either in the volumetric (left panel) or spherical (right panel) coordinate system. D) The group-average mean locations of the visual areas in left and right hemispheres. The average locations calculated in the volumetric coordinate system are marked with coloured squares and the white circles around the squares show the mean distance of the individual mean locations to the group average. The average locations calculated in the spherical coordinate system are marked with coloured circles and the mean distances with the black circles.

Figures 12A and 12B illustrate the difference between calculating the midpoint (centre-of-mass) of area V1 along the cortical surface in the spherical coordinates (Eq. 5) and in the volumetric 3D coordinates. After the volumetric averaging, the mean locations of V1 for the individuals spread on the lips of the calcarine sulcus (Fig. 12A), whereas after the spherical averaging, the points clustered at the base of the calcarine sulcus (Fig. 12B). Overall, the V1 loci for the 15 subjects were more widely distributed when the midpoint of the visual area was calculated in the volumetric coordinate space than when the calculation was done in the spherical cortical surface-based coordinates. To quantify the spread of the points, we calculated the distances of the individual V1 loci from their average location. The distances were significantly smaller, indicating tighter clustering, when the V1 midpoints were characterized in the spherical surface-based coordinates than when calculated in the volumetric coordinates (p<0.001, Wilcoxon Signed Rank-test across subjects; results from the two hemispheres averaged). This was true both when the distances from the individual loci to the average were compared in the 3D volumetric coordinate space (6.0 mm vs. 2.9 mm) and when compared along the 2D spherical cortical surface (5.1° vs. 2.3°; Eq. 7).

Figure 12C shows the mean locations of several visual areas for each individual on the average cortical surface. Overall, the spread of the visual area loci was reduced when the midpoints of the areas were calculated in the spherical coordinates compared to volumetric coordinates. This is summarized in Figure 12D, where the average locations of the visual areas are shown together with the average standard distances for both coordinate systems. The inter-individual variability in the visual area loci decreased when the midpoints of the visual areas were calculated along the spherical cortical surface and not in the conventional volumetric coordinate system (black vs. white circles in Fig. 12D).

**Figure 13:**
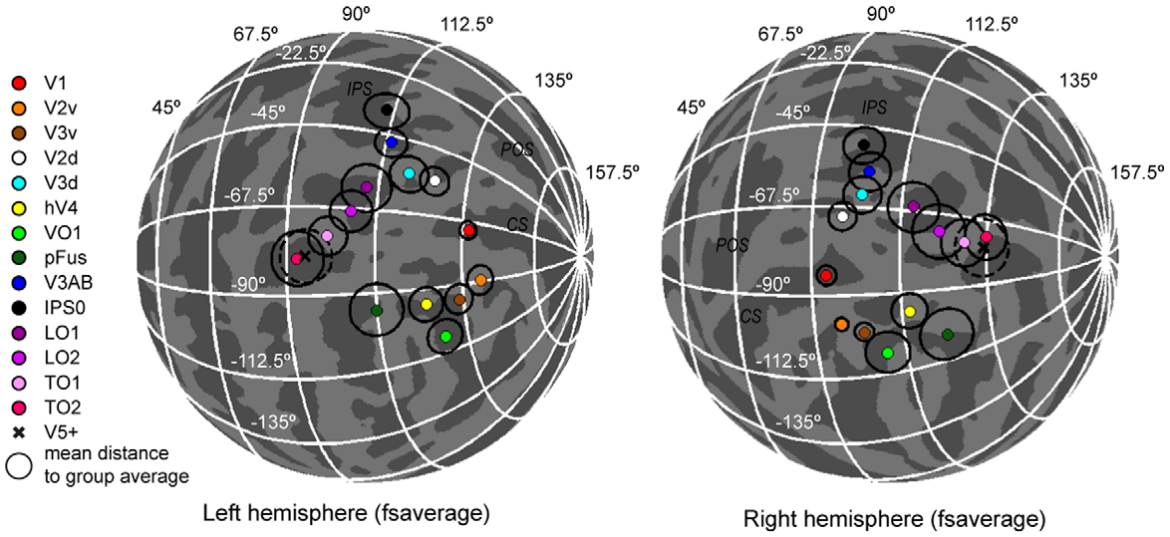
The mean locations of retinotopic visual areas on spherical cortical surface. The mean locations of several visual areas (see Table 1 for the coordinates) were calculated along the spherical cortical surface and are shown on the average spherical cortical surface of the left and right hemispheres of the Freesurfer's surface-based atlas (CS = Calcarine Sulcus; POS = Parieto-Occipital Sulcus; IPS = Intra-Parietal Sulcus). The black circles represent the average standard distances of the individuals' visual area locations from their mean. In addition to the retinotopic visual areas localized with the object mapping, the mean locations of visual area V5 is shown in both hemispheres with black crosses and the average standard distances with dashed circles.

The mean positions (computed in the spherical cortical surface-based coordinate system along the cortical surface) and the variability of the visual areas in the spherical surface coordinates are visualized in Figure 13 and listed in Table 1. In addition to the retinotopic visual areas localized with the object mapping (blocked design), we show the positions of the visual motion sensitive area V5 in both hemispheres. The longitude and latitude coordinates on the spherical surface-based atlas provide a compact representation of the visual area loci, where nearby coordinate values refer to nearby points on the cortical surface.

**Table 1:** Locations of visual areas in spherical surface-based coordinates.

	LEFT HEMISPHERE			RIGHT HEMISPHERE		
	latitude (°)	longitude (°)	st dist (°)	latitude (°)	longitude (°)	st dist (°)
**V1**	136.3	−70.7	2.2	94.0	−84.8	2.4
	[131 141]	[−72 −67]		[92 98]	[−87 −77]	
**V2v**	139.7	−89.2	3.6	97.1	−97.3	1.6
	[134 149]	[−99 −79]		[95 99]	[−101 −95]	
**V3v**	133.5	−94.8	3.7	102.6	−99.7	2.4
	[129 139]	[−104 −89]		[99 106]	[−104 −97]	
**V2d**	126.2	−56.2	3.7	97.9	−69.9	3.5
	[122 132]	[−69 −51]		[95 104]	[−75 −61]	
**V3d**	119.6	−55.7	4.8	102.4	−64.1	4.7
	[115 126]	[−68 −47]		[95 109]	[−71 −56]	
**hV4**	124.8	−94.7	4.3	114.3	−95.1	4.4
	[117 131]	[−100 −87]		[108 121]	[−102 −89]	
**VO1**	128.7	−106.4	4.5	107.7	−106.0	5.5
	[122 137]	[−115 −100]		[98 114]	[−112 −101]	
**pFus**	112.3	−94.6	6.6	123.0	−103.8	6.7
	[106 122]	[−109 −86]		[113 130]	[−113 −95]	
**V3AB**	113.7	−47.4	4.0	103.6	−57.7	5.1
	[109 120]	[−56 −40]		[94 114]	[−65 −52]	
**IPS0**	110.6	−37.4	5.6	101.1	−50.5	5.1
	[104 119]	[−43 −30]		[90 107]	[−57 −45]	
**LO1**	109.4	−61.6	6.1	115.5	−66.3	6.4
	[105 113]	[−72 −48]		[109 123]	[−75 −57]	
**LO2**	106.1	−68.2	5.2	122.3	−72.7	6.7
	[101 110]	[−78 −53]		[115 132]	[−92 −63]	
**TO1**	100.6	−74.9	4.8	128.7	−75.8	5.9
	[95 107]	[−82 −67]		[122 138]	[−95 −61]	
**TO2**	92.9	−80.6	6.7	134.6	−73.3	5.6
	[82 104]	[−87 −71]		[127 148]	[−84 −63]	
**V5**	95.0	−80.1	6.7	133.6	−77.1	7.1
	[82 107]	[−85 −72]		[127 141]	[−92 −63]	

Mean, range and average standard distance of visual area coordinates are listed in the spherical coordinate space for both hemispheres. In addition to the retinotopic visual areas localized with the object mapping, the coordinates are also given for the visual motion sensitive V5 complex. For a visualization of the visual area positions on the spherical cortical surface, see Figure 13.

### A surface-based probabilistic atlas of the retinotopic visual areas

**Figure 14:**
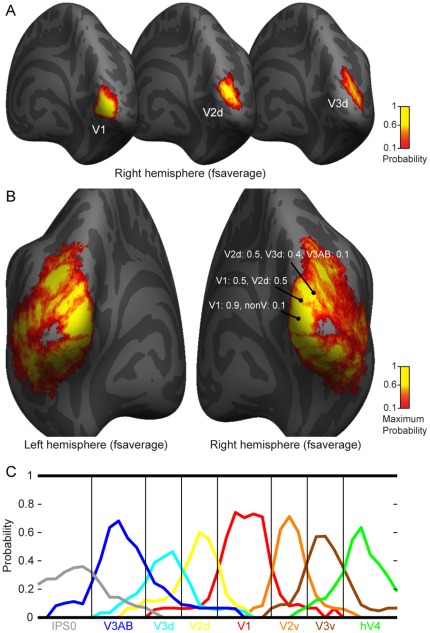
Surface-based probabilistic maps of the visual areas on Freesurfer's surface-based atlas of human cerebral cortex. A) Spatial probability maps of visual areas V1, V2d, and V3d on the average cortical surface. B) Maximum probability atlas of visual areas. The probabilities of different visual areas are shown for representative vertices as examples from the surface-based probabilistic atlas which can be obtained from the website http://ltl.aalto.fi/wiki/Atlas. C) Iso-polar line ROI analysis of the progression of the visual area probabilities along the cortical surface.

We constructed spatial probability maps of the retinotopic visual areas based on the data from the 15 subjects. Representative probability maps for visual areas V1, dorsal V2, and dorsal V3 in the right hemisphere are shown in Figure 14A. For each node in the average cortical surface, we counted the number of times the node was labelled as a specific visual area and divided this by the number of subjects. Figure 14B shows the maximum probability maps for the average left and right hemispheres, where the colour scale shows the maximum probability of a visual area in a specific cortical node. The probabilities peak at the centre of an area and decrease towards the borders between the areas. Figure 14C shows the progression of the visual area probabilities along the cortical surface. High probabilities especially within the early/mid-level visual areas suggest high predictability of the visual area locations based on the combination of the cortical curvature and topology information. The relatively low probability values in the lateral occipito-temporal cortex suggest low predictability of the LO/TO maps based on the cortical curvature information. The probability atlas of the retinotopic visual areas is available from the webpage http://ltl.aalto.fi/wiki/Atlas. The atlas comprises, for both hemispheres of the Freesurfer's fsaverage subject, the probability maps of the visual areas, the maximum probability maps and the annotation files of the probabilities of different visual areas in each vertex.

## Discussion

The retinotopic organization in V1, V2, V3, hV4, VO1, V3AB, IPS0, LO1, LO2, TO1, and TO2 could be defined based on data from a simple blocked fMRI design with object stimuli at different locations of the visual field. This result shows that a GLM-based mapping is a feasible alternative to phase-encoded retinotopic mapping. In addition, the 24-region multifocal stimulus was appropriate for the retinotopic mapping of the visual areas V1, V2, V3, hV4, and V3AB, and thus outperformed the original 60-region multifocal stimulus [24]. Overall this work provides an alternative to the phase-encoded retinotopic mapping especially for studies where the GLM analysis is preferred and the retinotopic organization is used as a functional localizer of multiple retinotopic regions-of-interest. The mapping tools can be obtained from the website http://ltl.aalto.fi/wiki/RetinotopicMapping. The object images in the blocked design and the checkerboards in the multifocal design would be easily replaced by any desired visual stimuli and could be combined with a task directed to the stimuli.

The use of a blocked stimulus design supported a straightforward characterization of the spatial tuning in different visual areas. The strength of the tuning decreased systematically across the hierarchy of visual areas, which likely reflected the increase in the average receptive field size, and more generally also the change from clearly retinotopic areas to object-responsive cortex where information is integrated across longer distances in the visual field [50]. The transitions in the tuning strength between visual areas clearly complement the visual area border information based solely on the retinotopic organization. This information could be particularly useful for defining the border between hV4/VO1 and pFus, or for separation of IPS0 or LO1 from the low/mid-level retinotopic areas.

We presented the mean and variability of the visual area positions in the spherical cortical surface-based coordinate system of the widely used Freesurfer software package [32], [42]. Inter-individual variability in the visual area loci decreased when the midpoints of the visual areas were calculated along the spherical cortical surface when compared to the conventional volumetric coordinate system. The results were collected also to probability maps of the retinotopic visual areas on Freesurfer's surface-based atlas of the human cerebral cortex (see http://ltl.aalto.fi/wiki/Atlas), which could be used as a reference for the functional organization and variability of the visual areas on the cortical surface.

### Multifocal mapping of retinotopic responses

The 24-region multifocal mapping was effective in the low/mid-level visual areas and within these areas the retinotopic maps were detailed. The multifocal mapping of the higher-level visual areas was not possible most likely due to the broad spatial tuning found in single voxels coupled with the pronounced nonlinear suppressive interactions between the stimulus regions [41], [48]. Our pilot experiments confirmed that the multifocal design was not suitable for higher-level visual areas even when fewer, larger stimulus regions were used and the checkerboards were replaced by images of objects. Thus, the more trivial explanation that the checkerboards are not optimal for the higher-level visual areas does not explain the reduction of the multifocal signals. This result suggests that higher-order visual activations for concurrently stimulated retinotopic representations cannot be recovered by linear analysis. It is likely that this non-linearity is not specific to retinotopic representations, suggesting more generally that concurrent activation patterns in the higher-level visual areas cannot be recovered by linear analysis methods.

Nonetheless, the 24-region multifocal stimulus outperformed the results obtained with the original 60-region multifocal stimulus [24], which was mainly appropriate for the mapping of the V1. The aforementioned nonlinear suppressive interactions between the multifocal regions in the low/mid-level visual areas [41] likely explain also the advantage of the 24-region stimulus over the 60-region multifocal stimulus. Compared to the 60-region stimulus, besides the fewer and larger stimulus regions in the 24-region stimulus, the concurrently stimulated regions were also divided into two temporally interleaved windows.

Multifocal stimulus design provides a straightforward analysis and interpretation of the retinotopic responses. The stimuli and the analysis scripts can be obtained from http://ltl.aalto.fi/wiki/RetinotopicMapping. The responses are analyzed with the standard general linear model implemented in all conventional fMRI software packages. In addition, any static or dynamic stimuli could be easily windowed to multifocal spatial and temporal design. Compared to the phase-encoded retinotopic approach, the multifocal method may also stand out in specific cases. For example, whereas the phase-encoded retinotopic stimuli are highly predictable, the spatial layout of the multifocal stimulus appears random to the subject, thus supporting fixation and enabling retinotopic behavioural paradigms. In pathological cases, where only part of the visual field is represented in the cortex, GLM methods may also provide more straightforward interpretation of the activation results.

In addition to retinotopic mapping, both mapping approaches are well suited as functional localizers of retinotopic regions-of-interest, which is not as straightforward with the phase-encoded retinotopic mapping. Moreover, multifocal mapping is more efficient than a randomized block design in localizing multiple retinotopic positions in the early visual areas [51]. On the other hand, for an efficient functional localizer, the number of multifocal regions can also be reduced to cover only the visual field positions that are relevant for the main experiment. Even a single experimental run measured in the beginning of an experiment can be enough to map multiple retinotopic ROIs in several visual areas (for an example, see [52]).

### Retinotopic organization in higher-level visual areas

The object mapping (blocked design) results were in good agreement with the previous phase-encoded retinotopic measurements showing full contralateral hemifield representations in hV4 and VO maps along the ventral cortex [9], [10], [13] and in LO1–2 and TO1–2 maps along the occipito-temporal cortex [18], [21]. To the authors' knowledge, this was the first time retinotopic organization in the occipito-temporal cortex was mapped with a blocked stimulus presentation. Previous blocked fMRI studies have only reported a bias for the contralateral visual field but no detailed retinotopic organization [26], [27]. The inclusion of face and place images in the stimulus set might enhance the responses in the areas anterior to VO2 along the parahippocampal cortex, but a high-resolution imaging protocol may also be needed [11].

We used a conventional voxel size in order to have a good signal-to-noise ratio and to cover a reasonably large part of the cortex. High-resolution fMRI might reveal retinotopic maps within the area we have labelled pFus. Consistent with studies on cortical processing of object stimuli [53], we found that the pFus region responded strongly to the object stimuli, but we could not find a retinotopic organization within this area that was consistent across the subjects. The human V5 complex, where our results agreed with the two hemifield maps (TO1 and TO2) [18], was recently divided to several distinct areas using high-resolution retinotopic mapping [20]. To map the visual field representation in IPS1–4 would likely require higher signal-to-noise ratio or a task that would engage subject's attention to the stimulated part of the visual field [15], [54], [55].

### Widening of the spatial tuning and ipsilateral responses in higher-level visual areas

Our measure of spatial tuning is closely related to the population receptive field (pRF) method developed by Dumoulin et al. [22]. In the pRF method, drifting bar stimuli evoke waves of fMRI responses along the retinotopic visual cortex. The centre and width of a circularly symmetric Gaussian model fitted to the fMRI responses describes the location and size of the pRF for each voxel. Here we did not assume any specific model for the response field in a voxel, but estimated the strength of the spatial tuning directly from the fMRI responses. Thus, our approach should provide unbiased estimates in areas where strong anisotropy may render the assumption of symmetric response biased. Our polar plots of spatial tuning (Figs. 7 and 8) suggest that the population receptive fields may be asymmetric, especially in cortical areas where the receptive fields extend to the ipsilateral visual field. Overall the gradual change in the spatial tuning along the hierarchy of visual areas is in good agreement with the results obtained with the pRF method [10], [18], [22]. Furthermore, our results suggest that the tuning strength could assist the identification of visual area borders based on retinotopic data, especially in the border between hV4 or VO maps and pFus, as well as between IPS0 or LO1 and the low/mid-level retinotopic areas.

We quantified the amount of ipsilateral responses in different visual areas and found consistent shift to more pronounced ipsilateral representations along the hierarchy of visual areas. Previously, fMRI activation studies [19], [27], [56] and more recently the pRF studies [18], [22] have reported responses for ipsilateral stimuli mainly in the higher-level visual areas. Our results extend the previous results on the ipsilateral visual field representations as they quantify the gradual increase in the responses for the ipsilateral stimuli along the hierarchy of visual areas. The V5 complex has been divided to areas MT/V5 and MST based on differences in the responses for moving stimuli in the peripheral ipsilateral visual field [19] and the same is true for retinotopic maps TO1 and TO2 that likely correspond to MT/V5 and MST [18]. The choice of relatively central stimuli (<8°) might have affected our results on the amount of ipsilateral responses in the visual areas with pronounced representation of the visual periphery, but the overall differences between visual areas likely reflected the amount of ipsilateral coverage of the receptive fields.

### Asymmetries in visual field representations

Behavioural studies indicate a better performance in the lower than in the upper visual field (for a review, see [57]). Consistent with this, we found the overrepresentation of the lower visual field compared to the representation of the upper visual field in areas LO1, LO2, pFus, V3AB, and TO1. Areas V1–3 also showed a tendency for the lower visual field bias, which is consistent with asymmetries in fMRI activation amplitude and extent in V1/V2 for stimuli on the lower and upper vertical meridian reported by Liu et al. [58]. Liu et al. emphasized, however, that this effect would be restricted to the upper vs. lower meridian. The lower visual field bias in the lateral occipital visual areas has been reported previously and is discussed in detail by Sayres and Grill-Spector [28]. Our finding of the overrepresentation of lower visual field in area pFus is in contrast to the result by Kravitz et al. [59], who reported an upper field bias within this area. The difference could be explained by different criterion in defining the pFus area, because there is yet no unambiguous definition for this cortical region [10] and we did find an upper field bias in the neighbouring ventral visual areas hV4 and VO1.

### Visual areas in cortical surface-based coordinate system

There is growing evidence that location on the cortical surface predicts the positions of visual areas more accurately than conventional stereotaxic coordinates such as Talairach or MNI [8], [38], [60], [61]. A coordinate system based on the cortical folding pattern would thus appear a natural choice to report locations of functional areas in the cortex. Such coordinate systems have been available for some time now [31], [62], but nevertheless the stereotaxic coordinates are still typically used in reporting functional area positions and activation loci. Here we reported the locations of the visual areas in the spherical surface-based coordinate system provided by the widely used Freesurfer software. We also showed that the inter-individual variability in the visual area loci decreased when the midpoints of the visual areas were calculated along the spherical cortical surface compared to the conventional volumetric coordinate system. Thus we would like to promote the use of surface-based coordinate system in reporting functional areas and activation loci. The longitude and latitude coordinates on the spherical cortical surface provide a coordinate space that respects the topology of the cortex and provide a concise description of the functional area positions [32], [36], [37]. Based on population data, it would even be possible to determine probability distributions for the area loci on the cortical surface and apply standard clinical approaches to determine whether the location of an area in a patient is outside 95% confidence interval. An alternative would be to develop and validate multivariate analysis methods for the comparison of functional area topology between individual and reference group data.

Group analysis of visual areas benefits from the cortical surface-based inter-individual alignment methods [31], [32]. Hagler et al. [63], [64] have developed methods for group analysis of the phase-encoded retinotopic mapping data and showed that the inter-individual variability in the phase-encoded retinotopic maps can be reduced via the alignment based on the sulcal anatomy. In our surface-based group-average maps, the retinotopic organization in the early/mid-level visual areas was well-preserved, but the retinotopic maps in the higher-level visual areas in the lateral occipito-temporal cortex showed considerable inter-subject variability, which resulted in blurring of the colours in the group retinotopic maps. This result confirms the relationship between the cortical folding and visual areas, which is strongest in V1 and weakens in the higher-level areas [21], [38], [60], [61]. Our results extend the previous studies by reporting the locations and variability of the visual areas in the cortical surface-based coordinates. In addition, our results suggest that there is more inter-individual variability in the orientation of the retinotopic maps in the lateral occipito-temporal cortex than, for example, in the ventral occipital cortex (Figs. 3 and 4).

Finally, we constructed the surface-based probabilistic maps of the visual areas on the Freesurfer's cortical surface atlas. Probabilistic maps or atlases of functional areas can be used as a reference for functional organization of the human visual cortex. Van Essen et al. [37] introduced the idea of probabilistic atlas of human visual cortex by generating average maps of eight visual areas (V1, V2, V3, VP, V3A, V4v, V8, MT+) based on data from four subjects. Hinds et al. [33], [38] showed that the location of V1 can be accurately predicted based on the individual's cortical folding and provided a high quality probability atlas of the V1. More recently, Yamamoto et al. [39], however, reported an average probability of only 0.27 for 12 retinotopic areas (V1, V2d/v, V3d/v, V3A, V3B, V7, LOc, MT, V4v, V8) on the cortical surface with a slightly higher probability for visual area V1. In our opinion, the average probability is not a very good measure of the inter-individual alignment of visual areas, because the probability peaks in the middle of a visual area and drops towards the border between areas. Based on our results, we would argue that the overall consistency of visual areas on the cortical surface is much higher than is generally assumed.

### Conclusions

We showed that retinotopic organization in several visual areas could be mapped with a simple blocked fMRI design. Multifocal mapping with the 24-region stimulus was suitable for retinotopic mapping of the visual areas V1–V3AB/hV4. Retinotopic mapping in each individual is currently the best approach for the localization of visual areas. That said the probability maps of the areas and the average coordinates on a cortical surface-based atlas brain provide an overview of the locations and variability of the visual areas and may also help in situations where individual retinotopic maps are not available or are incomplete. We would like to encourage researchers to publish their surface-based group-analysis data and the coordinates for future meta-analysis of, *e.g.*, functional area and activation loci in the surface-based coordinate system.

## Supporting Information

Video S1 **A 30-second video excerpt of the multifocal stimulus.**
(WMV)

Video S2 **A 30-second video excerpt of the object stimulus.**
(WMV)

Figure S1 **Pilot experiments with multifocal fMRI targeting the retinotopic mapping of higher-level visual areas.** Two subjects (S16, S17) participated in several pilot fMRI mapping experiments, in which we tested different spatial and temporal parameters of the multifocal stimulus. Representative results from these experiments are shown here (A–E). For a reference, the bottom row (F) shows the polar angle maps obtained with the object mapping (blocked design, 9 stimulus regions) used in the main experiments. A) Polar angle maps obtained with a 9-region multifocal stimulus. B) Polar angle maps obtained with a 5-region multifocal stimulus. C) Polar angle maps obtained with a 5-region multifocal stimulus with images of objects within the stimulus regions. D) Polar angle maps obtained with a 5-region multifocal stimulus with natural images (van Hateren JH, van der Schaaf A (1998) Independent component filters of natural images compared with simple cells in primary visual cortex. Proc Biol Sci 265: 359–366.) within the stimulus regions. E) Polar angle maps obtained with a 5-region multifocal stimulus with natural images within the stimulus regions, and during one miniblock, active stimulus regions were displayed consecutively to reduce suppressive interactions between the regions. F) Polar angle maps obtained with the object mapping stimulus (Figures 1C–D). Maps were constructed from two runs of object mapping data to have comparable amounts of data as in A–E.
(TIF)

Figure S2 **Eccentricity and polar angle maps obtained with the multifocal stimuli for the right hemisphere for 15 subjects.**
(TIF)

Figure S3 **Eccentricity and polar angle maps obtained with the multifocal stimuli for the left hemisphere for 15 subjects.**
(TIF)

Figure S4 **Eccentricity and polar angle maps obtained with the object stimuli for the right hemisphere for 15 subjects.**
(TIF)

Figure S5 **Eccentricity and polar angle maps obtained with the object stimuli for the left hemisphere for 15 subjects.**
(TIF)

Figure S6 **Retinotopic organization of ventral visual cortex mapped with the object stimuli.** Ventral views of the retinotopic eccentricity and polar angle maps on the right hemisphere for all 15 subjects.
(TIF)

Figure S7 **Retinotopic organization of ventral visual cortex. Same as in Supplementary Figure S6 for the left hemisphere.**
(TIF)

Figure S8 **Retinotopic organization of lateral visual cortex mapped with the object stimuli.** Lateral views of the retinotopic eccentricity and polar angle maps on the right hemisphere for all 15 subjects. The third panel shows the cortical areas that are sensitive to visual motion.
(TIF)

Figure S9 **Retinotopic organization of lateral visual cortex. Same as in Supplementary Figure S8 for the left hemisphere.**
(TIF)

Figure S10 **Cortical maps of polar angle tuning strength for both hemispheres for all 15 subjects.**
(TIF)
