# Supplementary figures and images for: Retinotopic Maps, Spatial Tuning, and Locations of Human Visual Areas in Surface Coordinates Characterized with Multifocal and Blocked fMRI Designs
Source: PLoS One. 2012 May 9;7(5):e36859. doi: 10.1371/journal.pone.0036859 (PMC3348898; doi:10.1371/journal.pone.0036859)

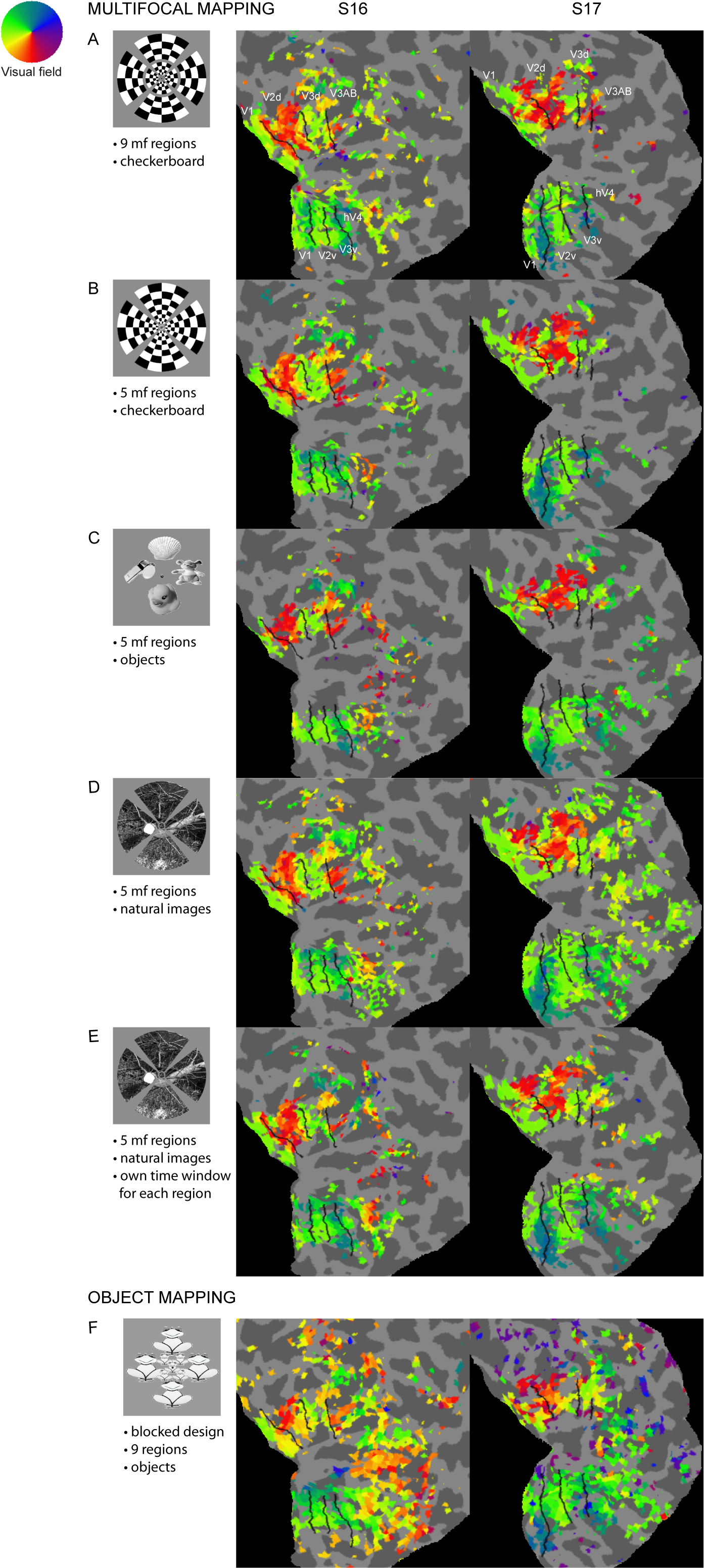

Supplement: Figure S1 — Pilot experiments with multifocal fMRI targeting the retinotopic mapping of higher-level visual areas. Two subjects (S16, S17) participated in several pilot fMRI mapping experiments, in which we tested different spatial and temporal parameters of the multifocal stimulus. Representative results from these experiments are shown here (A–E). For a reference, the bottom row (F) shows the polar angle maps obtained with the object mapping (blocked design, 9 stimulus regions) used in the main experiments. A) Polar angle maps obtained with a 9-region multifocal stimulus. B) Polar angle maps obtained with a 5-region multifocal stimulus. C) Polar angle maps obtained with a 5-region multifocal stimulus with images of objects within the stimulus regions. D) Polar angle maps obtained with a 5-region multifocal stimulus with natural images (van Hateren JH, van der Schaaf A (1998) Independent component filters of natural images compared with simple cells in primary visual cortex. Proc Biol Sci 265: 359–366.) within the stimulus regions. E) Polar angle maps obtained with a 5-region multifocal stimulus with natural images within the stimulus regions, and during one miniblock, active stimulus regions were displayed consecutively to reduce suppressive interactions between the regions. F) Polar angle maps obtained with the object mapping stimulus (Figures 1C–D). Maps were constructed from two runs of object mapping data to have comparable amounts of data as in A–E. (TIF) [file pone.0036859.s003.tif]

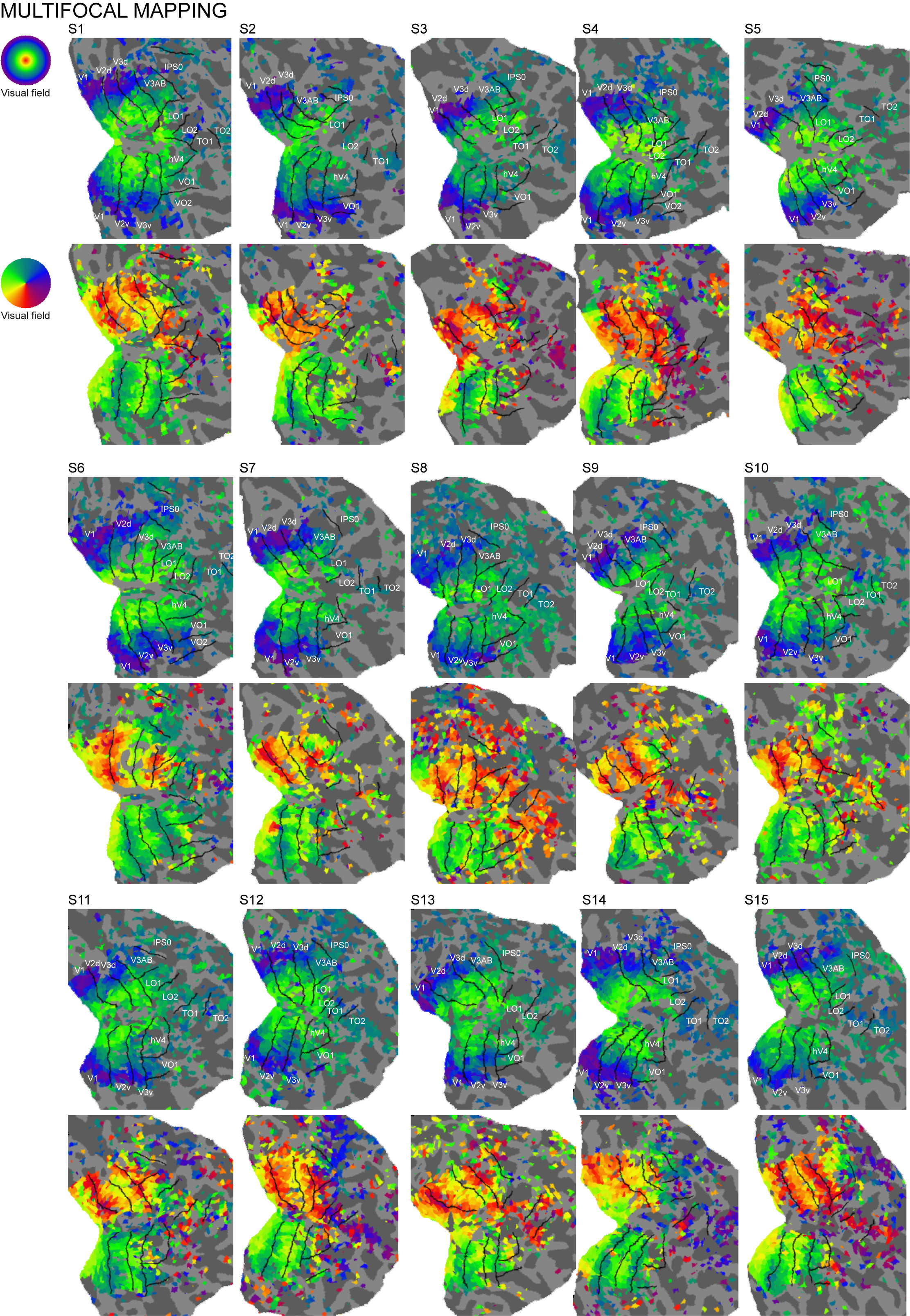

Supplement: Figure S2 — Eccentricity and polar angle maps obtained with the multifocal stimuli for the right hemisphere for 15 subjects. (TIF) [file pone.0036859.s004.tif]

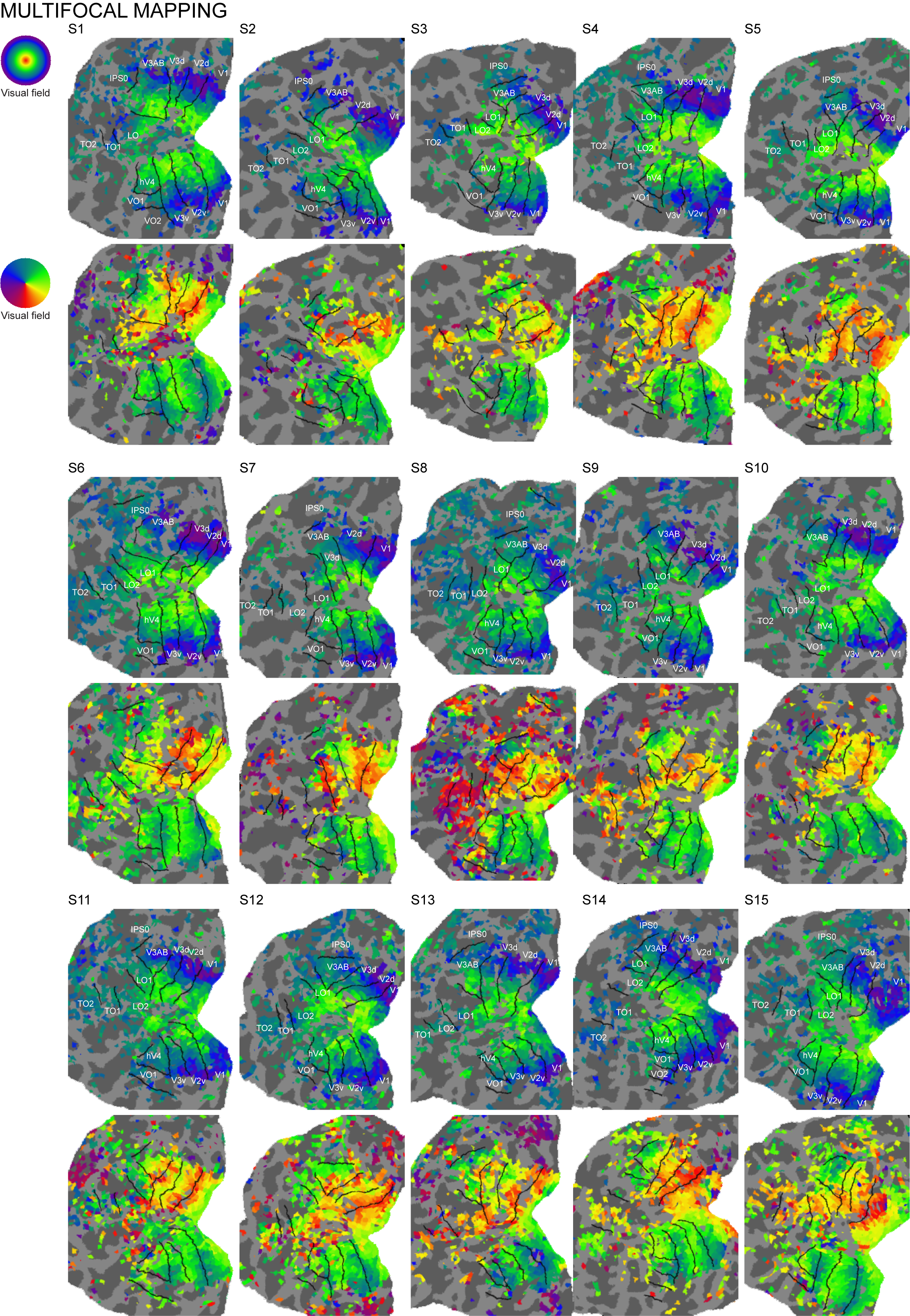

Supplement: Figure S3 — Eccentricity and polar angle maps obtained with the multifocal stimuli for the left hemisphere for 15 subjects. (TIF) [file pone.0036859.s005.tif]

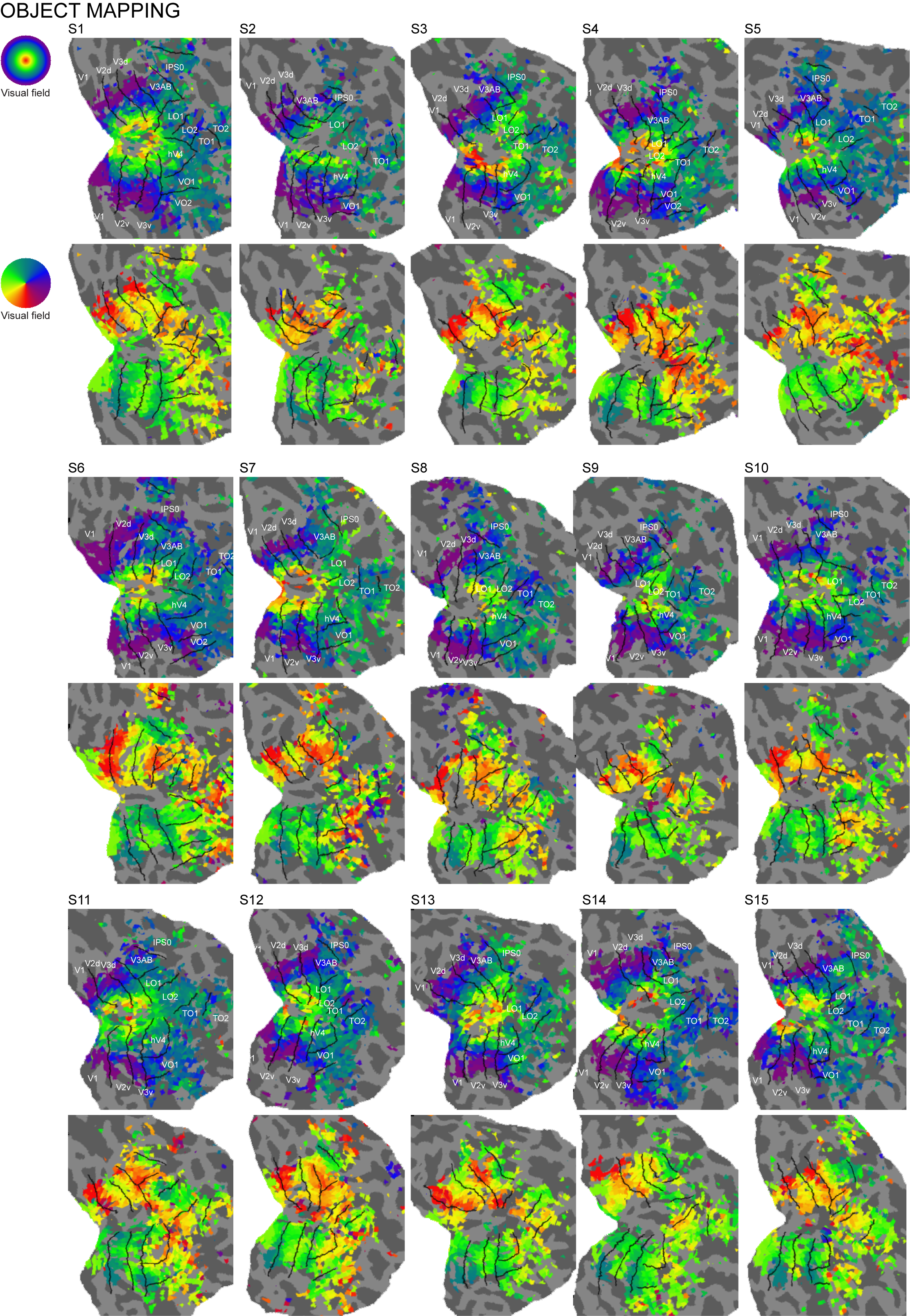

Supplement: Figure S4 — Eccentricity and polar angle maps obtained with the object stimuli for the right hemisphere for 15 subjects. (TIF) [file pone.0036859.s006.tif]

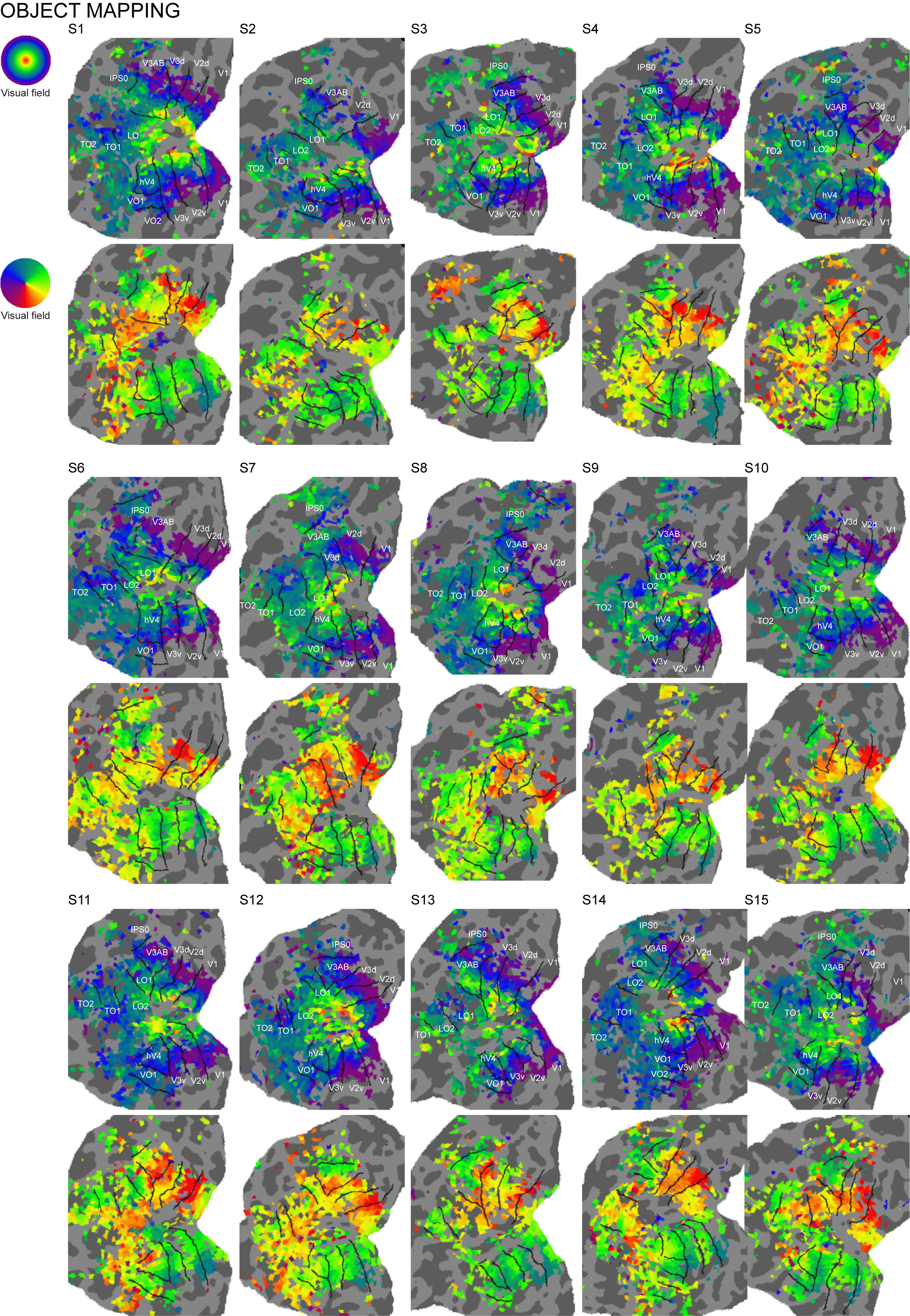

Supplement: Figure S5 — Eccentricity and polar angle maps obtained with the object stimuli for the left hemisphere for 15 subjects. (TIF) [file pone.0036859.s007.tif]

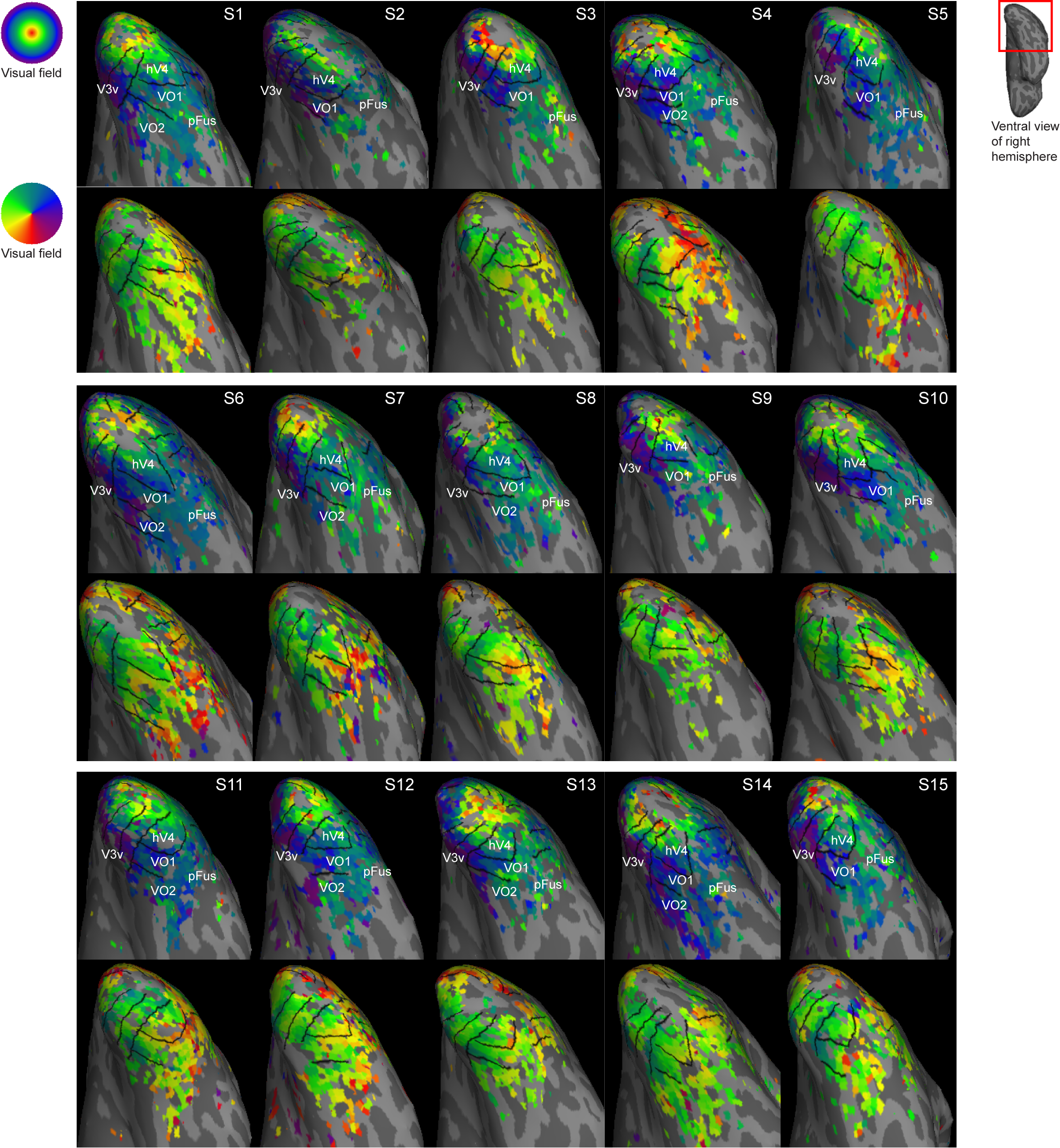

Supplement: Figure S6 — Retinotopic organization of ventral visual cortex mapped with the object stimuli. Ventral views of the retinotopic eccentricity and polar angle maps on the right hemisphere for all 15 subjects. (TIF) [file pone.0036859.s008.tif]

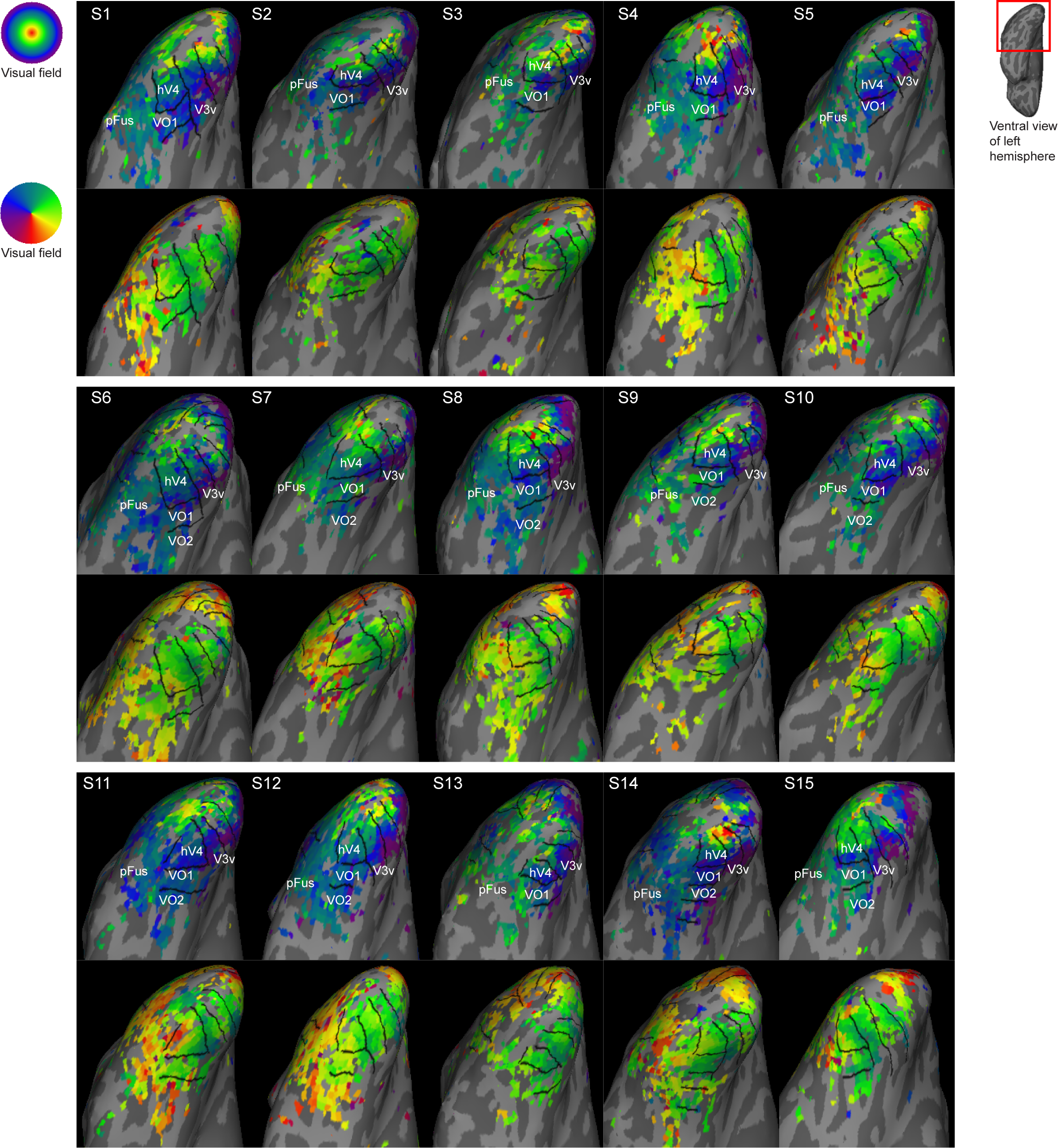

Supplement: Figure S7 — Retinotopic organization of ventral visual cortex. Same as in Supplementary Figure S6 for the left hemisphere. (TIF) [file pone.0036859.s009.tif]

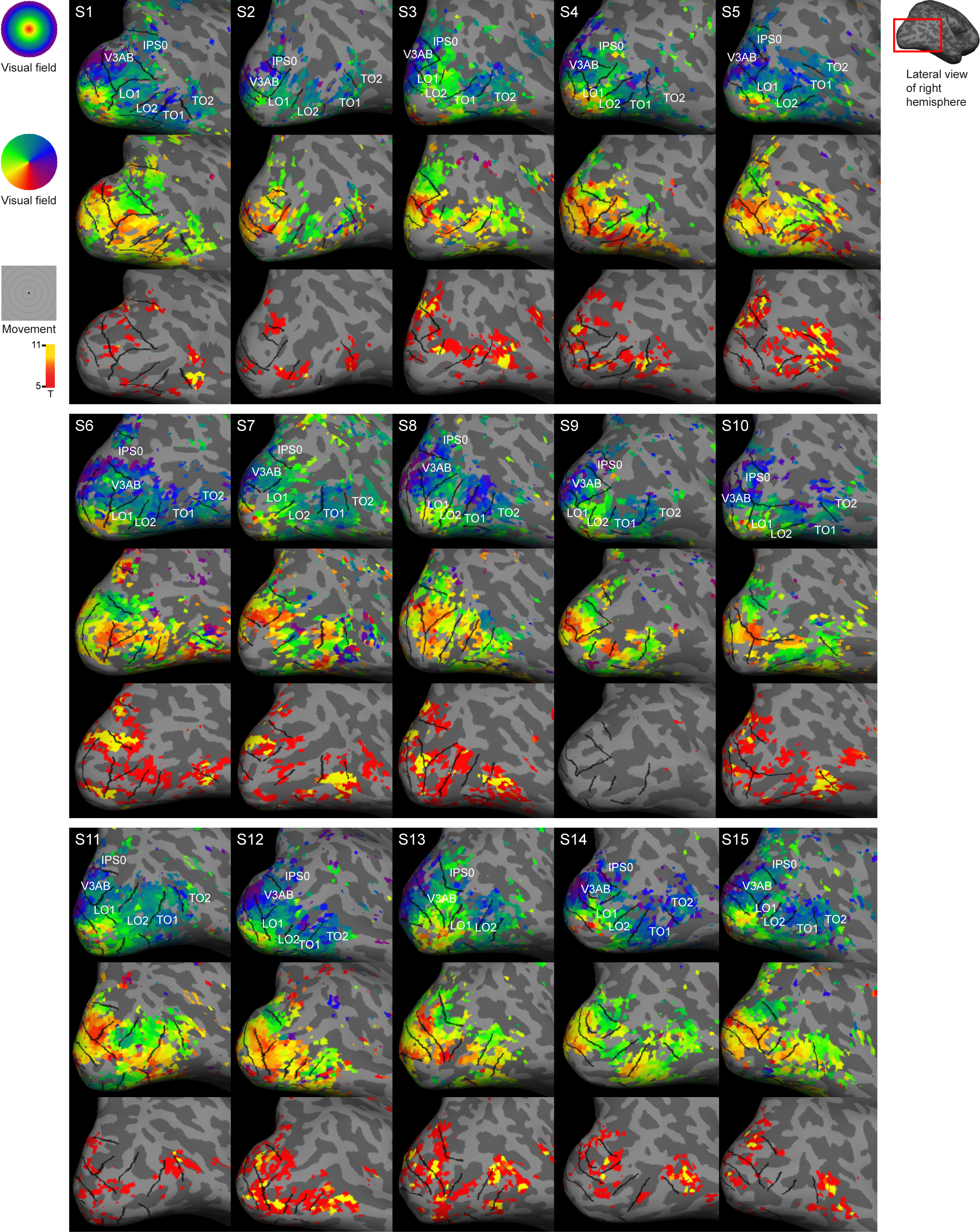

Supplement: Figure S8 — Retinotopic organization of lateral visual cortex mapped with the object stimuli. Lateral views of the retinotopic eccentricity and polar angle maps on the right hemisphere for all 15 subjects. The third panel shows the cortical areas that are sensitive to visual motion. (TIF) [file pone.0036859.s010.tif]

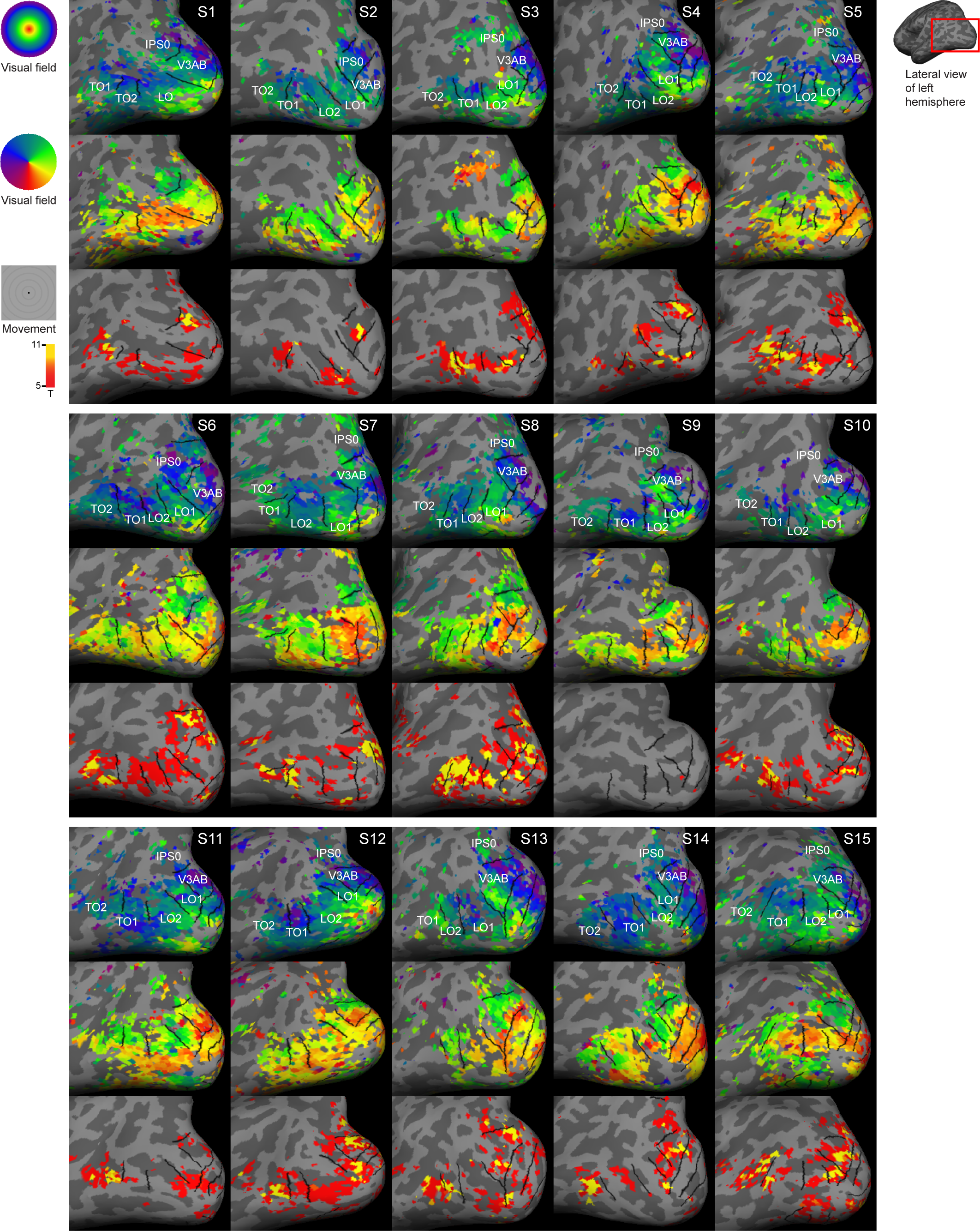

Supplement: Figure S9 — Retinotopic organization of lateral visual cortex. Same as in Supplementary Figure S8 for the left hemisphere. (TIF) [file pone.0036859.s011.tif]

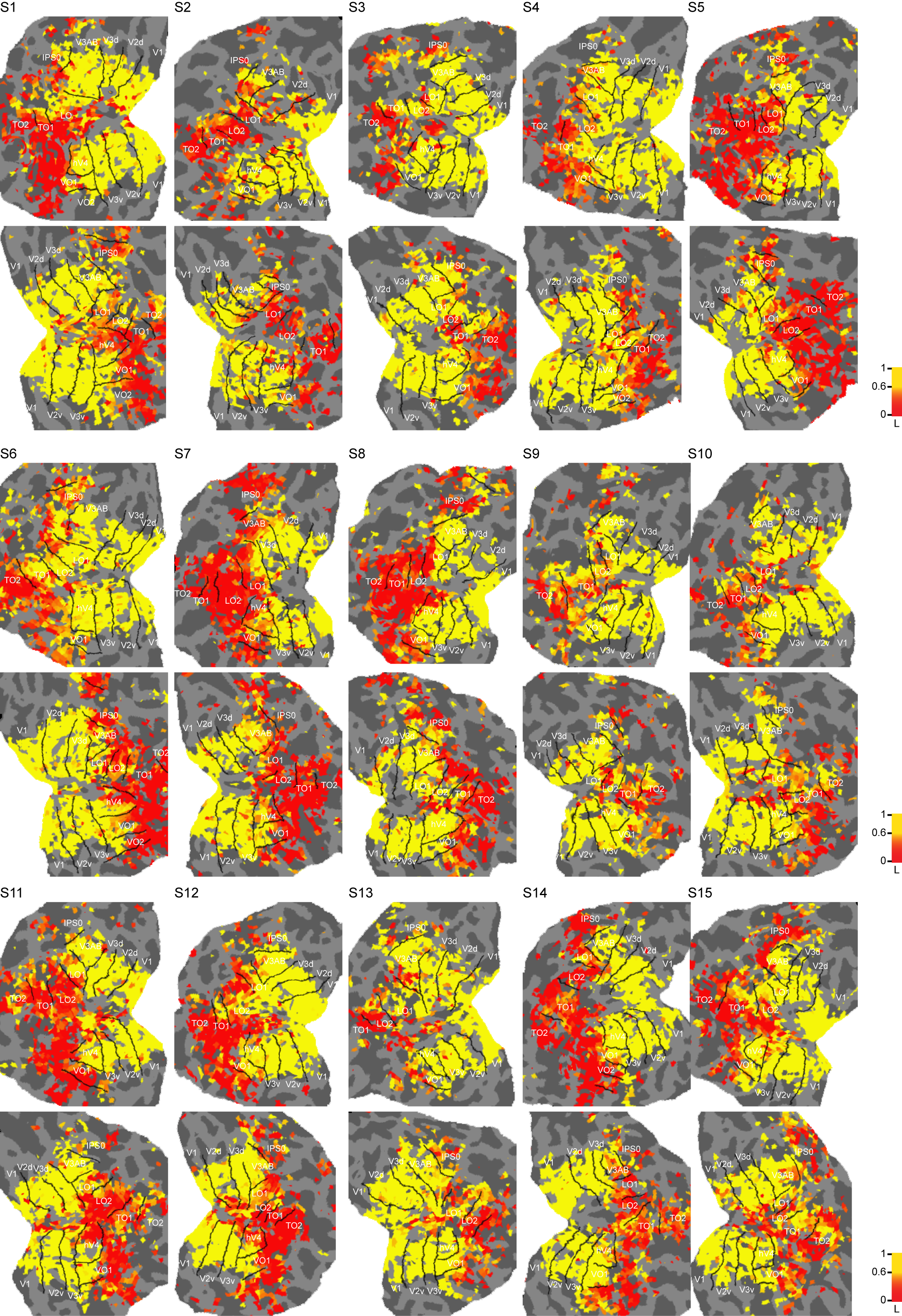

Supplement: Figure S10 — Cortical maps of polar angle tuning strength for both hemispheres for all 15 subjects. (TIF) [file pone.0036859.s012.tif]
